# Cortical neuronal hyperexcitability and synaptic changes in *SGCE* mutation-positive myoclonus dystonia

**DOI:** 10.1093/brain/awac365

**Published:** 2022-10-07

**Authors:** Alessandra Sperandeo, Claudia Tamburini, Zoe Noakes, Daniel Cabezas de la Fuente, Francesca Keefe, Olena Petter, William Plumbly, Nicholas E Clifton, Meng Li, Kathryn J Peall

**Affiliations:** Neuroscience and Mental Health Research Institute, Division of Psychological Medicine and Clinical Neuroscience, Cardiff University, Hadyn Ellis Building, Cardiff CF24 4HQ, UK; Neuroscience and Mental Health Research Institute, Division of Psychological Medicine and Clinical Neuroscience, Cardiff University, Hadyn Ellis Building, Cardiff CF24 4HQ, UK; Neuroscience and Mental Health Research Institute, Division of Psychological Medicine and Clinical Neuroscience, Cardiff University, Hadyn Ellis Building, Cardiff CF24 4HQ, UK; Neuroscience and Mental Health Research Institute, Division of Psychological Medicine and Clinical Neuroscience, Cardiff University, Hadyn Ellis Building, Cardiff CF24 4HQ, UK; Neuroscience and Mental Health Research Institute, Division of Psychological Medicine and Clinical Neuroscience, Cardiff University, Hadyn Ellis Building, Cardiff CF24 4HQ, UK; Neuroscience and Mental Health Research Institute, Division of Psychological Medicine and Clinical Neuroscience, Cardiff University, Hadyn Ellis Building, Cardiff CF24 4HQ, UK; Neuroscience and Mental Health Research Institute, Division of Psychological Medicine and Clinical Neuroscience, Cardiff University, Hadyn Ellis Building, Cardiff CF24 4HQ, UK; Neuroscience and Mental Health Research Institute, Division of Psychological Medicine and Clinical Neuroscience, Cardiff University, Hadyn Ellis Building, Cardiff CF24 4HQ, UK; Neuroscience and Mental Health Research Institute, Division of Psychological Medicine and Clinical Neuroscience, Cardiff University, Hadyn Ellis Building, Cardiff CF24 4HQ, UK; Neuroscience and Mental Health Research Institute, Division of Psychological Medicine and Clinical Neuroscience, Cardiff University, Hadyn Ellis Building, Cardiff CF24 4HQ, UK

**Keywords:** dystonia, neurodevelopment, cortical neurons, hyperexcitability, synaptic transmission

## Abstract

Myoclonus dystonia is a childhood-onset hyperkinetic movement disorder with a combined motor and psychiatric phenotype. It represents one of the few autosomal dominant inherited dystonic disorders and is caused by mutations in the ε-sarcoglycan (*SGCE*) gene. Work to date suggests that dystonia is caused by disruption of neuronal networks, principally basal ganglia-cerebello-thalamo-cortical circuits. Investigation of cortical involvement has primarily focused on disruption to interneuron inhibitory activity, rather than the excitatory activity of cortical pyramidal neurons. Here, we have sought to examine excitatory cortical glutamatergic activity using two approaches: the CRISPR/Cas9 editing of a human embryonic cell line, generating an *SGCE* compound heterozygous mutation, and three patient-derived induced pluripotent stem cell lines, each gene edited to generate matched wild-type *SGCE* control lines. Differentiation towards a cortical neuronal phenotype demonstrated no significant differences in either early- (PAX6, FOXG1) or late-stage (CTIP2, TBR1) neurodevelopmental markers. However, functional characterization using Ca^2+^ imaging and microelectrode array approaches identified an increase in network activity, while single-cell patch clamp studies found a greater propensity towards action potential generation with larger amplitudes and shorter half-widths associated with *SGCE* mutations. Bulk RNA sequencing analysis identified gene ontological enrichment for ‘neuron projection development’, ‘synaptic signalling’ and ‘synaptic transmission’. Examination of dendritic morphology found *SGCE* mutations to be associated with a significantly higher number of branches and longer branch lengths, together with longer ion-channel dense axon initial segments, particularly towards the latter stages of differentiation (Days 80 and 100). Gene expression and protein quantification of key synaptic proteins (synaptophysin, synapsin and PSD95), AMPA and NMDA receptor subunits found no significant differences between the *SGCE* mutation and matched wild-type lines. By contrast, significant changes to synaptic adhesion molecule expression were identified, namely higher presynaptic neurexin-1 and lower postsynaptic neuroligin-4 levels in the *SGCE* mutation carrying lines. Our study demonstrates an increased intrinsic excitability of cortical glutamatergic neuronal cells in the context of *SGCE* mutations, coupled with a more complex neurite morphology and disruption to synaptic adhesion molecules. These changes potentially represent key components to the development of the hyperkinetic clinical phenotype observed in myoclonus dystonia, as well a central feature to the wider spectrum of dystonic disorders, potentially providing targets for future therapeutic development.

## Teaser

Sperandeo *et al.* show that cortical stem cell models of *SGCE*-mutation positive myoclonus dystonia demonstrate a hyperexcitable phenotype at both network and single cell level. Gene expression analysis reveals longer and more complex neurite morphology, and disruption of synaptic adhesion molecules.

## Introduction

Dystonia is one of the most common forms of movement disorder, with an estimated population prevalence of 1.2%.^1^ It involves loss of coordinated contraction of antagonistic muscle groups, leading to abnormal postures and pain, impacting quality of life.^2^ As well as representing a primary disorder, dystonia is also a phenotypic component of many neurodevelopmental and neurodegenerative disorders.^3,4^ In excess of 25 Mendelian inherited, dystonia-causing genes have now been identified, with these predominantly resulting in the early-onset motor symptoms.^5^ One such disorder is myoclonus dystonia, caused by mutations in the autosomal dominantly inherited ε-sarcoglycan (*SGCE*) gene, encoding the ε-sarcoglycan protein, and whose penetrance is reduced owing to maternal imprinting.^6,7^ The clinical phenotype typically involves cervical and/or upper limb dystonia, an upper body predominant myoclonus and pronounced psychiatric phenotype.^8,9^ The ε-sarcoglycan protein is a single-pass transmembrane glycoprotein,^10^ expressed embryonically and postnatally, suggesting its importance in development.^11^ Recent purification of the brain-specific form of ε-sarcoglycan suggests it forms part of a brain-specific dystrophin-associated protein complex,^12^ with ultra-deep sequencing of post-mortem brain tissue demonstrating high levels of expression in the primary somatosensory cortex.^13^

Understanding of dystonia pathophysiology is limited, although evidence from human imaging, murine and post-mortem studies indicate that it is caused by disruption of neuronal networks, principally the basal ganglia-cerebello-thalamo-cortical circuits.^14^ Several studies have demonstrated a central role for the cerebral cortex within these networks, with a recent unbiased genetic-systems biology approach reinforcing the importance of frontal cortical neurons in dystonia.^15^ Human imaging studies further support cortical involvement with multiple modalities identifying larger somatosensory and grey matter volumes, impaired sensorimotor functional connectivity and sensorimotor white matter microstructural changes in those affected with dystonia.^16–20^ Neurophysiological studies also indicate the importance of the cortex in dystonia with murine models demonstrating increased long-term potentiation (LTP) and reduced long-term depression (LTD), prominent at cortico-striatal synapses.^21,22^ Similar features have been identified in human studies of focal dystonias noting increased LTP-like and LTD-like plasticity of the motor cortex.^23^ Several studies have suggested that impaired cortical surround inhibition is responsible for these changes^24^; however, more recent work has suggested a more complex picture with factors other than reduced inhibition contributing to the hyperexcitable phenotype.^25^

To begin to probe the specific cellular changes contributing to the network-based pathophysiology in dystonia, and more specifically the contribution of disrupted cortical excitability, we sought to develop a myoclonus dystonia model via CRISPR-edited embryonic stem cells and patient-derived induced pluripotent stem cells (iPSC). Through differentiation towards an excitatory cortical glutamatergic lineage, we examine the impact of loss of ε-sarcoglycan expression on cortical neuronal development and function.

## Materials and methods

### Experimental model

#### Induced pluripotent stem cell lines and participation recruitment

Participants with NHS laboratory-confirmed *SGCE* mutations were recruited via the Welsh Movement Disorders Research Network, giving signed informed consent to derivation of iPSC lines in line with the Declaration of Helsinki (REC for Wales, IRAS ID: 146495, REC ref: 14/WA/0017). All participants were examined and confirmed to have a clinical phenotype consistent with that of myoclonus dystonia.

#### Culture, reprogramming and characterization of primary lymphoblasts

Whole blood samples were collected by venepuncture in EDTA tubes. Peripheral blood mononuclear cells (PBMC) were isolated with Lymphoprep and Sepmate tubes according to STEMCELL Technologies protocol. Isolated PBMCs were reprogrammed with a CytoTune™-iPS 2.0 Sendai Reprogramming Kit (Invitrogen). PBMC were then activated using cytokines SCF, FLT-3, IL-3 and IL-6 for 4 days. Transductions were performed with CytoTune™ 2.0 Sendai viruses, removed after 24 h. Cells were cultured for the next 2 days in StemPro™-34 SFM medium with cytokines. On Day 3, cells were placed on Gibco™ Mouse Embryonic Fibroblasts and maintained in culture until Day 8 when the media was replaced with iPSC medium. Wells were monitored for the emergence of iPSC colonies, with Tra1-60 positive colonies manually picked and expanded. Colonies were stained using anti-SeV antibodies until all were negative. The absence of the CytoTune™ 2.0 Sendai reprogramming vectors was confirmed by RT-PCR.

#### Generation of induced pluripotent stem cell lines with *SGCE* mutation correction

CRISPR/Cas9 correction, through generation of double strand breaks by homology-directed repair (HDR), of all patient-derived reprogrammed cell lines [labelled Pt1, Pt1 Corrected (Pt1C), Pt2, Pt2C, Pt3, Pt3C] was outsourced to Applied Stem Cell (www.appliedstemcell.com). Sanger sequencing (Supplementary Fig. 1) and copy number variation (CNV) analysis was performed on receipt to confirm correction to wild-type sequence and absence of off-target effects (Table 1).

**Table 1:** Clinical and genetic characteristics of patient-derived stem cells and gene edited embryonic cell line

Patient-derived cell line										
		Clinical characteristics			Genetic characteristics					
	Sex	Age at onset	Motor symptoms	Non-motor symptoms	*SGCE* mutation	CNV Analysis				
						Patient line			Corrected line	
						Blood	iPSC	Neuronal	iPSC	Neuronal
Patient 1 (Pt1)	Female	2.5 years	Myoclonus: UL, T Dystonia: N, UL	D, PanD, SP, GAD	Exon 6 c.771_772delAT, p.Cys258X	2q37.3 150 kb Del	2q37.3 150 kb Del	2q37.3 150 kb Del	2q37.3 150 kb Del	2q37.3 150 kb Del
Patient 2 (Pt2)	Female	7 years	Myoclonus: UL, T Dystonia: N, UL	Nil	Exon 5 c.622G > A, p.Gly221Asp	2q14.3 232.5 kb Dup 12p13.31 120 kb Dup	2q14.3 232.5 kb Dup 12p13.31 120 kb Dup	2q14.3 232.5 kb Dup 12p13.31 120 kb Dup	2q14.3 232.5 kb Dup 12p13.31 120 kb Dup	2q14.3 232.5 kb Dup 12p13.31 120 kb Dup
Patient 3 (Pt3)	Male	3 years	Myoclonus: N, UL, T Dystonia: N, UL	D, SP, OCD	Exon 5 c.622G>A, p.Gly221Asp	No CNV detected	No CNV detected	No CNV detected	No CNV detected	No CNV detected
**Human embryonic cell line**						***SGCE*ko**			**Wild-type**	
hESC							**PSC**	**Neuronal**	**PSC**	**Neuronal**
	–	–	–	–	–	–	7q11.23 320 kb Dup 8p23.2 239 kb Dup	7q11.23 320 kb Dup 8p23.2 239 kb Dup	7q11.23 320 kb Dup 8p23.2 239 kb Dup	7q11.23 320 kb Dup 8p23.2 239 kb Dup

Detailed motor and non-motor clinical characteristics of the three patient-derived stem cell lines (Patients 1–3), together with CNV analysis to demonstrate lack of off-target effects during CRISPR/Cas9 gene editing nor accumulation of additional CNVs during neuronal differentiation process. Where possible, this analysis was undertaken in patient blood samples, following reprogramming to pluripotent status and following differentiation towards a cortical neuronal lineage. CNV analysis also undertaken in the gene edited hESC line, both pre- and post-neuronal differentiation. D = depression; Del = deletion; Dup = duplication; GAD = generalized anxiety disorder; N = neck; PanD = panic disorder; SP = social phobia; T = trunk; UL = upper limbs; (−) = not applicable.

#### Generation of *SGCE*ko human embryonic stem cell lines

The human embryonic stem cell (hESC) line, iCas9, was used to derive the *SGCE* null model, via non-homologous end joining.^26^ CRISPR guide RNAs (gRNAs) were designed using online tools (www.crispr.mit.edu) and selected based on lowest off-target predictive score. gRNAs were generated using *in vitro* transcription and amplified by PCR. Following purification (MiniElute PCR purification kit, Qiagen) the product was used as a template for *in vitro* transcription using the MEGAshortscript T7 transcription kit (Thermo Fisher), followed by purification (MEGAclear transcription clean up kit, Thermo Fisher). Doxycycline (2 µg/ml) was added at culture Day −1 to induce Cas9 protein expression, and Lipofectamine^®^ RNAiMAX (Thermo Fisher) used for gRNA transfection, with two consecutive transfections at 24-h intervals. Two days following the second gRNA transfection, iCas9 cells were dissociated and seeded into 10 cm dishes at densities of 500, 1000 and 2000 cells/dish. Colonies were mechanically picked when large enough, disaggregated, and plated into duplicate wells of 96 well plates containing Essential 8™ Flex medium and RevitaCell.

#### Genotyping and CNV calling

All genotyping data were generated by the Illumina iScan array using either the Infinium Global Screening Array v3.0 (patient and corrected lines) or the Infinium Psych Array v1.1 (*SGCE*wt and *SGCE*ko). The iScan data were uploaded to the Illumina Genome Studio v2.0.4 and call rate threshold set at 0.95. Datasets were analysed using the PennCNV software (http://penncnv.openbioinformatics.org/en/latest/). Quality controls steps included merging of CNVs with between CNV fraction of <0.2 (<20%), and exclusion of those <100 000 bp in length and containing <10 single nucleotide polymorphisms (SNPs). Log R ratio and B allele frequency plots were generated for each CNV and checked for reliability of the call.

### Experimental methods

#### Maintenance of human pluripotent stem cells and neural differentiation

**Figure 1:**
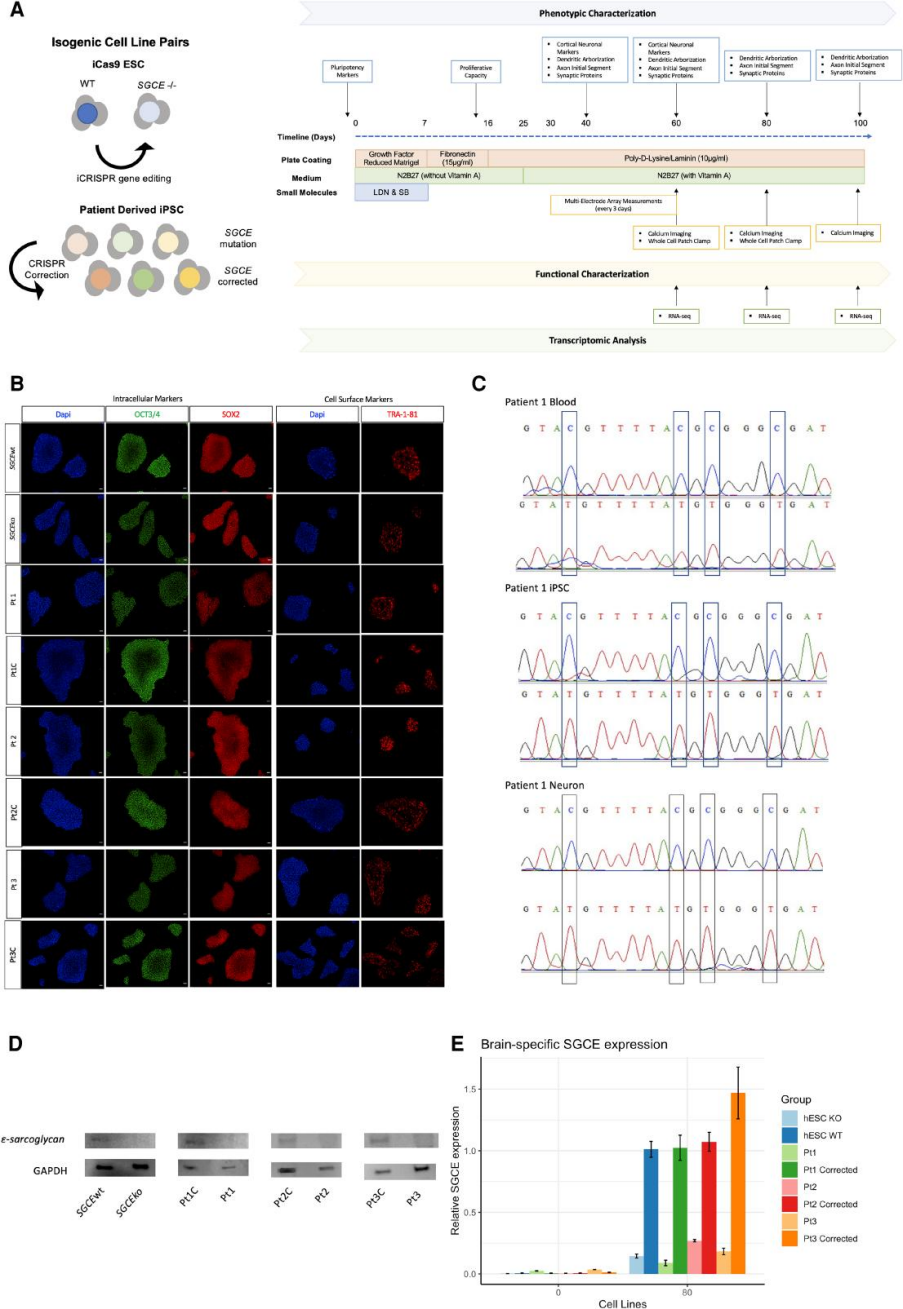
**Stem cell model derivation**. (**A**) Schematic overview of study design. *SGCE* mutant lines were derived using two approaches (i) CRISPR editing of the iCas9 human embryonic line; and (ii) reprogramming and genetic correction of unrelated patient-derived lines (*n* = 3, Pt1–3). The lines subsequently underwent phenotypic, function and gene expression characterization over a 100-day differentiation protocol towards an excitatory cortical glutamatergic lineage. (**B**) Representative immunofluorescence images for intracellular pluripotency markers—OCT1/4 and SOX2—and cell surface marker—TRA-1–81 demonstrating appropriate expression in the reprogrammed lines. Scale bars = 50 mm. (**C**) Exemplar of direct sequencing traces demonstrating the preservation of imprinting in the patient derived lines following blood sample collection, cellular reprogramming to a pluripotent state and differentiation towards a neuronal lineage. (**D**) Representative immunoblot for ε-sarcoglycan and loading control (GAPDH), demonstrating the absence of ε-sarcoglycan expression in all *SGCE* mutation carrying lines. (**E**) Quantitative RT-PCR for ε-sarcoglycan expression at Days 0 and 80. Each *SGCE* mutation carrying line is compared to their wild-type isogenic control. Data are presented as mean ± SEM from three independent experiments per line. Lines compared using one-way ANOVA analysis, with *P < 0.05. **P < 0.01, ***P < 0.001.

Human pluripotent stem cells were cultured on Matrigel^®^ (Corning, VWR International) and maintained in Essential 8™ Flex medium (Thermo Fisher) under standard culture conditions (37°C, 5% CO_2_). Stem cell media was changed on alternate days and cells passaged every 3–4 days when 70–80% confluency was reached. For cortical neuronal differentiation, cells were passaged onto growth factor reduced Matrigel (Corning, VWR), and grown to 80–90% confluency in Essential 8™ Flex. Medium was changed to N2B27 (without vitamin A), with this day designated Day 0 and followed previously published protocols.^27,28^ An overview of the differentiation protocol is provided in Fig. 1A.

#### Immunocytochemistry and imaging

Cultured cells were washed with PBS, fixed in cold 3.7% PFA for 15 min and re-washed with DPBS prior to staining. PBS-T (0.3% Triton-X-100 in PBS) was used to permeabilize cells for 10 min, followed by a 30-min block with 2% bovine serum albumin and 5% donkey serum (Gentaur). Cells were incubated overnight with primary antibodies in PBS-T with 5% donkey serum at 4°C. The subsequent day, cells were washed with PBS-T and incubated in the dark for 2 h with secondary antibodies (AlexaFluor anti-donkey 488, 555, 647; Life Technologies) diluted in PBS-T at room temperature. DAPI (Sigma), diluted 1:3000 in PBS, was used for nuclear staining. Stained cells were imaged using a Leica DM6000B inverted microscope, an average of 10 random fields of view for each staining combination at ×20 magnification. Cells were counted either manually (cytoplasmic markers) or using automated Cell Profiler (nuclear markers). N-cadherin was used for apical localization of the neural rosettes, with rosette size measured manually using FIJI (ImageJ). Immunocytochemistry quantification was collected from at least three biological replicates from at least three independent experiments for each marker. Antibodies are listed in the Supplementary material and Supplementary Methods Table 1.

#### Quantitative real-time PCR

Total RNA was extracted using TRI reagent, treated with TURBO DNase and cDNA generated [EvoScript Universal cDNA Master (Roche)]. Quantitative real-time PCR (qPCR) was performed using Mesa Green qPCR master mix to quantify genes of interest. Bio-Rad CFX Connect Real-Time System was used for the standardized qPCR programme (incubation at 95°C for 4 min, 40 cycles of 94°C for 30 s, 60°C for 15 s and 72°C for 30 s). Dissociation curves were recorded to check for amplification specificity. Data were analysed using the ΔΔ-CT method for relative quantification, with all data normalized to two reference genes (*GAPDH* and *ACTB* (β-Actin).^29,30^ Reactions were performed in triplicate for each cDNA sample, for each of the three differentiations. Primers are listed in Supplementary Methods Table 2.

#### Western blot analysis

Total protein was extracted from cultured cells using 1 × RIPA buffer (New England Biolabs) with added MS-SAFE Protease and Phosphotase Inhbitor (Sigma). Following quantification, equal amounts of each sample were loaded into a 4–12% Bolt^®^ Bis-Tris gel (ThermoFisher), separated and transferred to a methanol activated PVDF membrane (Amersham Hybond, GE Healthcare) via electro-blotting. Bovine serum albumin (5%) in Tris buffered saline containing 0.1% Tween (TBS-T) was used to block the membrane before incubating with primary antibodies overnight at 4°C. Following washing (×3) in TBS-T the subsequent day, secondary antibodies conjugated to horse-radish peroxidase (HRP) (Abcam) were applied and incubated for 1 h at room temperature. Secondary antibodies were detected following 3-min incubation with Luminata Crescendo HRP substrate (Millipore). Chemoluminescent detection was carried out using the iBright CL1000 (ThermoFisher). Images were exported to ImageJ for quantification.

#### Calcium imaging

Cultured cells were seeded onto poly-d-lysine/laminin-coated 13 mm glass coverslips (Days 15–20). On the day of recording, neurons were loaded with the calcium indicator Fluo-4AM (5 μM, Life Technologies) with 0.02% Pluronic F-127 (Life Technologies) and 0.01% Kolliphor EL (Sigma) for 1 h at 37°C. Artificial CSF (aCSF) was applied to cells and returned to the 37°C incubator for 30 min. Prior to recording, aCSF was replaced with fresh recording solution. An LED system (Rapp OptoElectronic and Lumencor) recorded calcium activity at 10 Hz for 10 min, with a final resolution of 1024 × 1024 pixels. Each time frame was split into four approximately equal regions of interest [Supplementary Fig. 4A(i and ii)]. For each region of interest, traces of fluorescence intensity over time were created and used as substrate for subsequent analysis [Supplementary Fig. 4B(i–iii)]. Fluorescence traces were normalized to the initial fluorescence intensity (F/F_0_) and the calcium transient parameters were calculated for active cells. Active cells were defined as cells showing at least three calcium transients within the measured period. A standalone MATLAB package, NeuroCa,^31^ was used to perform segmentation. The segmentation files were run in FluoroSNNAP, a MATLAB script for the analysis of calcium activity.^32^

#### Electrophysiology

Neural progenitors were seeded onto poly-d-lysine/laminin-coated glass coverslips, as described above. On the day of recording, coverslips were transferred to a recording chamber on an Olympus BX61W (Olympus) differential interference contrast (DIC) microscope and perfused at 2.5 ml/min with aCSF. Recordings were performed using a MultiClamp 700B amplifier and pipettes with resistances of 4–8 MW when filled with an intracellular recording solution. Electrophysiological data were sampled at 20 kHz and filtered at 3 kHz using a Digidata 1550 analogue to digital converter and pClamp 10 software (Molecular Devices). Series resistance was compensated using the bridge-balance and varied <20% during recordings. Resting membrane potential (RMP), input resistance (RN), membrane time constant (τ) and membrane capacitance (Cm) were measured in current clamp mode. For evoked activity measurement, cells were injected with current steps of 20 pA between −60 and +120 pA (1 s) to measure voltage responses. The properties of single action potentials were calculated from the first spike evoked by injecting a 5 ms pulse of 200 pA current. Data were analysed using Clampfit 10 software (Molecular Devices).

#### Microelectrode array measurements

Cells were plated onto poly-d-lysine/laminin-coated plates Cytoview microelectrode array (MEA) 24-well plates (Axion Biosystems) at D30 differentiation (75 000 cells/cm^2^). Extracellular recordings were obtained with an Axion Maestro system controlled by AxIS software (Axion Biosystems) with a 12.5 Hz sampling rate. Each well of the 24-well plate contained 16 electrodes/well to record spontaneous electrical activity. Recordings were performed at 37°C. Spontaneous activity was measured on every second day (Days 35–63).

#### Bulk RNA sequencing

Total RNA was collected using TRI reagent and extracted using the phenol/chloroform method. PureLink™ RNA Mini Kit column (Ambion), used according to manufacturer’s instructions, was used to remove any residual gDNA. Library preparation and sequencing were performed by Novogene (UK) Company Ltd. The RNA sample was used for library preparation using NEB Next^®^ Ultra RNA Library Prep Kit for Illumina^®^. Libraries were sequenced using an Illumina NovaSeq 6000 S4 flowcell with paired end 150 bp reads. Raw sequencing reads were trimmed using Trimmomatic (v0.38) with the SLIDINGWINDOW method and a filter for low quality (phred score <15) reads. Resulting reads were subjected to quality control by FastQC and aligned to the human reference genome (GRCh38) with STAR software. Duplicate reads were removed using Picard tool, MarkDuplicates (https://broadinstitute.github.io/picard/). Processed reads were mapped to genes with featureCounts (v.1.6.4) excluding duplicates, multimapping reads and chimeric fragments. Differential gene expression analysis of protein-coding genes was performed using DESeq2 (v3.14), for three samples from each human embryonic stem cell (hESC) line (*SGCE*wt and *SGCE*ko) at all three time points (Days 60, 80 and 100). Differentially expressed genes (DEGs) with an adjusted (false discovery rate, FDR) *P*-value <0.05 and absolute fold change >2 were considered statistically significant. Heat maps were generated from the row-scaled *z*-score of DEGs normalized counts obtained by DESeq2 with complete-linkage Euclidean hierarchical clustering. Gene ontology (GO) enrichment analyses were performed using enrichGO, using a background of all expressed protein-coding genes and Benjamini–Hochberg *P*-value correction of FDR <0.05 for statistical significance. Enrichment maps were generated from the enrichGO output data using emapplot function in R.

#### Neurite morphology analysis

Cortical projection neurons were analysed at Days 40, 60, 80 and 100. Seventy-two hours prior to assessment, cells were transfected with 500 ng pmaxGFP (Lonza) per well using Lipofectamine 3000 reagent (Thermo Fisher). Neuronal cultures were incubated with DNA-lipid complexes for 6 h at 37°C, then washed and cultured for a further 72 h. Neurons were fixed with 3.7% paraformaldehyde for 20 min, then stained with an anti-GFP antibody. GFP-positive neurons were visualized using a Leica DMI6000B inverted microscope and their morphology assessed using FIJI (ImageJ) with the semi-automated plugin Simple Neurite Tracer.

#### Statistical analysis

All analyses were done with R software (version 4.0.1), except ΔΔ-CT method for qPCR relative quantification (Microsoft Excel). One-way ANOVA analysis was used to test for significance during cross-sectional comparison of individual cell line pairs (case-control) and two-way ANOVA analysis used for case-control comparison across multiple time points. Mean values ± standard error of the mean (SEM) are shown, unless otherwise stated. Statistical significance was considered as *P*-values <0.05, and significances were represented as ^∗^*P* < 0.05, ^∗∗^*P* < 0.01 and ^∗∗∗^*P* < 0.001. Data presented are of four independent cell line pairs, over three differentiations, unless otherwise stated.

### Data availability

Data supporting the findings of this study are available from the corresponding author, upon reasonable request.

## Results

### *SGCE* mutations result in no significant changes to cortical glutamatergic neuronal developmental markers

**Figure 2:**
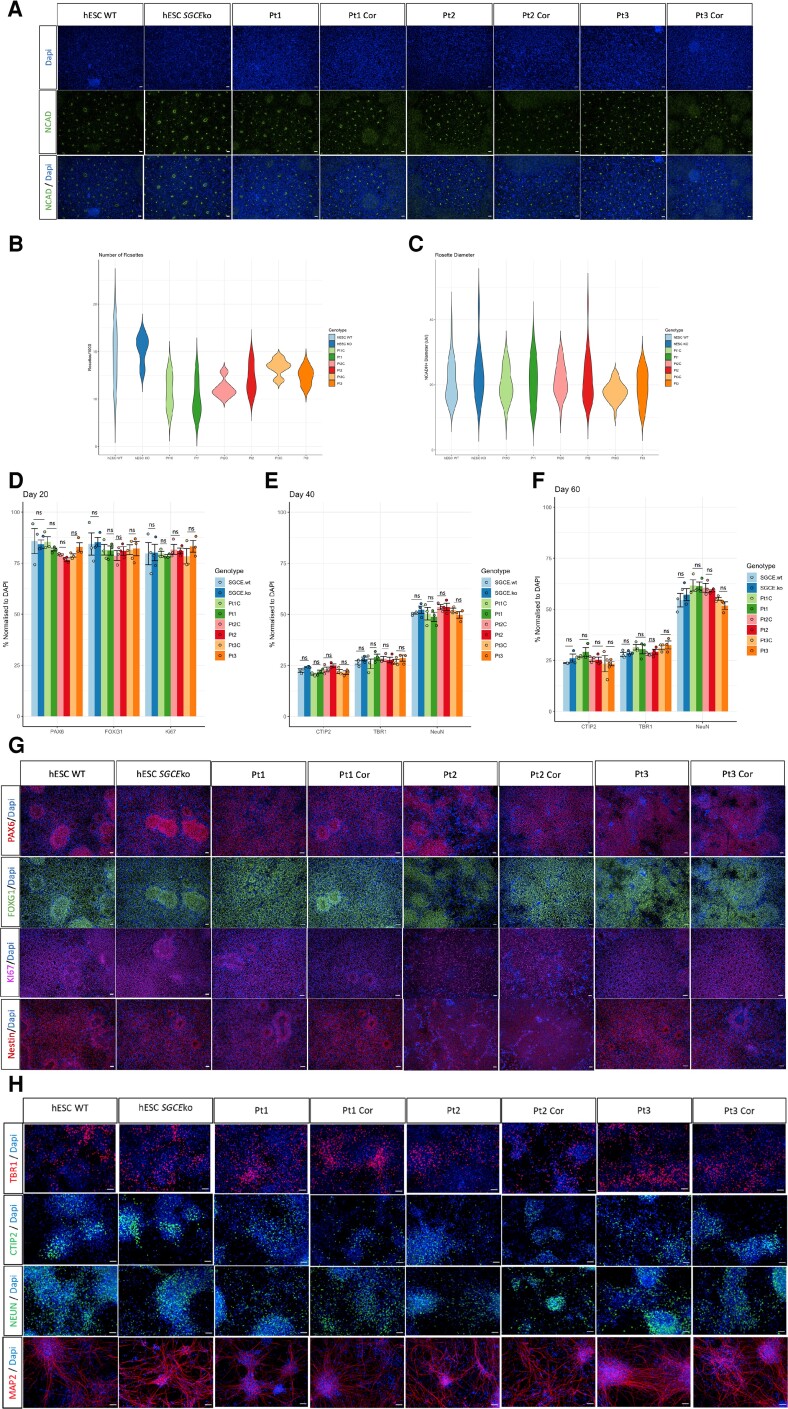
**Examination of cortical developmental markers**. (**A**) Representative immunofluorescence images for neural rosette marker, N-cadherin (NCAD) and counterstained with DAPI at Day 15 of differentiation. Scale bars = 50 mm. (**B** and **C**) Violin plots comparing the number of NCAD^+^ rosettes (**G**) and their diameter (**H**) between *SGCE* mutation carrying lines and their isogenic wild-type controls. Pairs of lines statistically compared using one-way ANOVA analysis. ns = not significant. (**D**–**F**) Quantification of immunofluorescent markers of neural progenitor status (**I**) and regional appropriate markers of differentiation (**J** and **K**) at Days 20 (**I**), 40 (**J**) and 60 (**K**) of differentiation. Isogenic matched lines compared using one-way ANOVA analysis at each time point. ns = not significant. (**G**) Representative immunofluorescence images of neural progenitor markers (PAX6, FOXG1, Ki67 and Nestin) at Day 20 differentiation. (**H**) Representative immunofluorescence images of cortical neuronal markers (TBR1, CTIP2, NEUN and MAP2) at Day 60 differentiation.

Four isogenic cell lines pairs, harbouring an *SGCE* mutation or wild-type sequence, were used to investigate the impact of loss of ε-sarcoglycan expression on cortical glutamatergic neuronal development and function (Fig. 1A). The first involved CRISPR editing of the iCas9 hESC line, producing an *SGCE* exon 2 compound heterozygous mutation (Supplementary Fig. 1A), hereafter referred to as the *SGCE*wt and *SGCE*ko lines, respectively.^33^ Biallelic *SGCE* mutations were required to mirror the autosomal dominant paternal inheritance pattern, combined with maternal imprinting, observed in the clinical genotype, with both mutations generated considered to be deleterious with *in silico* analysis (Supplementary Fig. 1B). Blood samples from three unrelated *SGCE*-mutation-positive patients with a clinical phenotype consistent with myoclonus dystonia were used to generate iPSC (Table 1), which were then edited via CRISPR/Cas9 to correct the mutations to wild-type state, generating an isogenic matched control for each line (Supplementary Fig. 1C–E). Immunohistochemistry of intracellular (OCT3/4, SOX2) and cell surface (TRA-1-81) pluripotency markers was consistently expressed across all eight cell lines (Fig. 1B), with all lines demonstrating their propensity to be able to differentiate towards each of the germ cell lines (Supplementary Fig. 2E). To ensure that the patient-derived iPSC models were a faithful representation of the clinical genotype, preservation of imprinting was confirmed in the initially collected DNA sample, following lymphoblastic cell line reprogramming, and differentiation towards a cortical neuronal lineage (Fig. 1C and Supplementary Fig. 2A–C). In addition, SNP analysis confirmed the absence of introduction of off-target CNVs during the gene editing, reprogramming or differentiation process (Table 1). Sanger sequencing across the target *SGCE* exon demonstrated no smaller deleterious variants in the proximity of the active gene editing window (Supplementary Fig. 1C–E). Protein expression analysis (Fig. 1D and Supplementary Fig. 2D) and quantitative gene expression analysis (Fig. 1E) at Day 80 differentiation, confirmed reduction in ε-sarcoglycan expression across all *SGCE* mutation carrying lines.^34^ Immunohistochemical staining of N-cadherin (NCAD) at Day 15 differentiation demonstrated that loss of ε-sarcoglycan expression had no impact on the formation and development of neural rosettes, with no significant difference in the number or diameter of the rosettes (Fig. 2A–C). By Day 20, both hESC and iPSC cultures expressed markers of pan- and dorsal-forebrain progenitors (PAX6, FOXG1), and ongoing proliferative capacity (NESTIN, Ki67), with no significant differences between wild-type and mutant cell line pairs in marker abundance (Fig. 2D and G and Supplementary Fig. 3A–C). Similarly, deep layer cortical identity proteins (CTIP2, TBR1) and neuronal markers (NEUN, MAP2) demonstrated comparable levels of immunohistochemical staining across all cell lines at Days 40 and 60 differentiation (Fig. 2E–H). These results were further supported by transcript analysis of cortical glutamatergic neuronal marker genes (*CTIP2*, *TUJ1*, *TBR1*, *SATB2*; Supplementary Fig. 3D–G), demonstrating no significant differences between the cell lines in each isogenic matched pair.

### Loss of ε-sarcoglycan expression results in higher levels of neuronal functional activity

**Figure 3:**
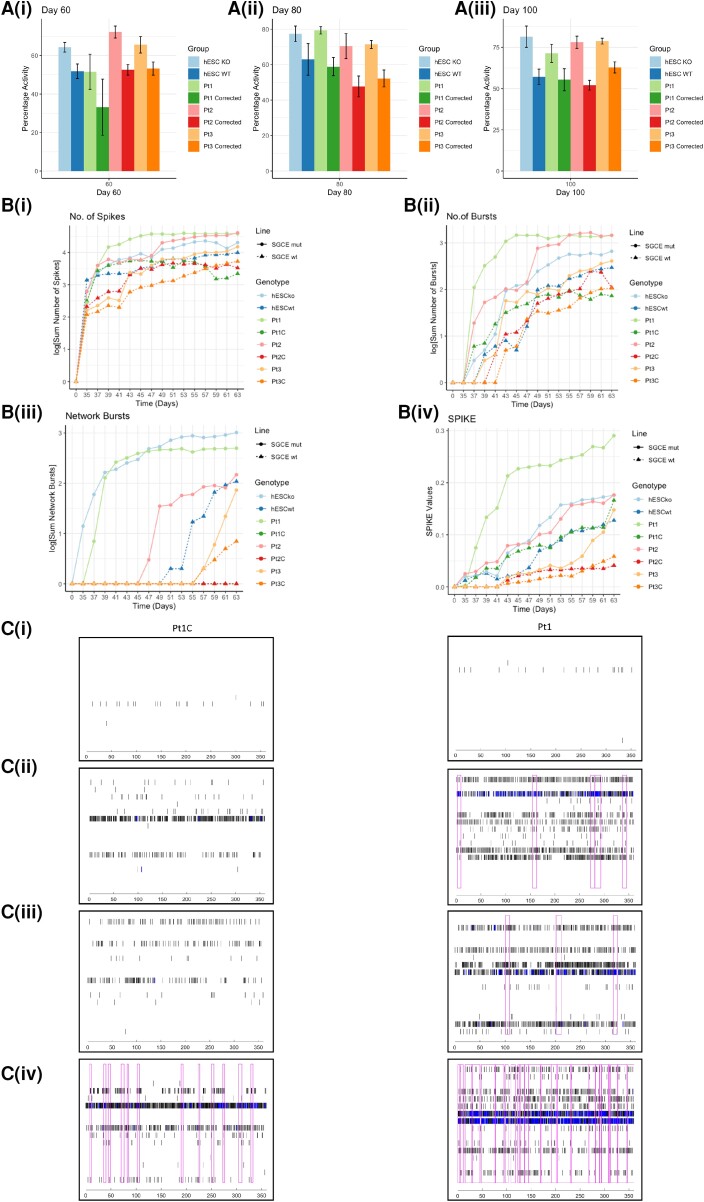
**Network based increased neuronal excitability.** (**A**) Overall cellular calcium activity measured as percentage of total number of active cells at Days 60 (**i**), 80 (**ii**) and 100 (**iii**) differentiation. Each *SGCE* mutation carrying line is compared to their wild-type isogenic control. Data are presented as mean ± SEM from three independent experiments per line. Lines compared using one-way ANOVA analysis, with *P < 0.05, **P < 0.01, ***P < 0.001. (**B**) MEA measurements taken on alternate days for 28 days (Days 35–63), including total number of spikes (**i**), bursts (**ii**), network bursts (**iii**) and SPIKE synchrony measurement (**iv**). Data are presented as line plots, with each point representing the mean value across 12 wells per experiment, from three independent experiments per line. Lines compared using two-way ANOVA analysis, with *P < 0.05. **P < 0.01, ***P < 0.001. Asterisks used only at time points where significant differences was observed across all cell line pairs, and least significant value used to determine asterisk labelling. (**C**) A comparison of representative raster plots from lines Pt1C and Pt1 at Day 35 (**i**), Day 45 (**ii**), Day 55 (**iii**) and Day 63 (**iv**). Vertical black bars represent individual spikes, blue shaded areas represent spike bursts and magenta highlighted areas represent network bursts. Time (ms) on the *x*-axis, extending from 0–360 ms.

**Figure 4:**
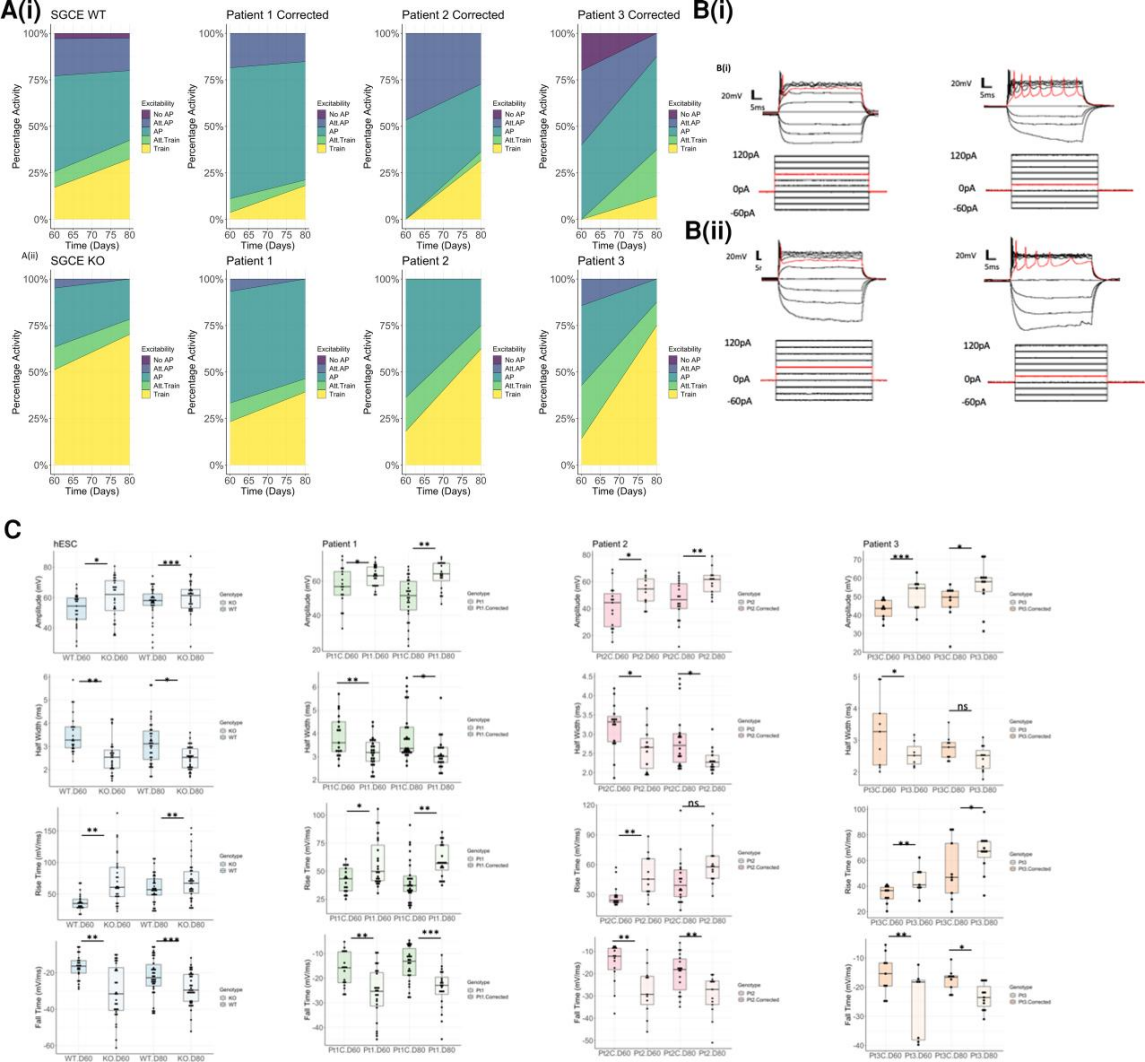
**Whole-call patch clamp meaurements.** (**A**) Stacked area graph of wild-type lines (**i**) compared to those harbouring *SGCE* mutations (**ii**), demonstrating increased overall excitability in the mutant lines with higher numbers of APs, attempted AP trains and AP trains, with these proportionally increasing between Days 60 and 80 of differentiation. (**B**) Schematic example of a representative trace of evoked activity resulting from current injection steps in the (**i**) *SGCE* WT and (**ii**) SGCEko human embryonic lines, and (**iii**) Pt1C corrected and (**iv**) Pt1 lines. (**C**) Box plots demonstrating AP characteristics that were significantly different across all paired cell lines at both Day 60 and Day 80 time points. These included larger amplitude, smaller half-width, faster rise and fall times in the *SGCE* mutation carrying lines, with these values increasing between Days 60 and 80 of differentiation. Lines compared using one-way ANOVA analysis, with *P < 0.05. **P < 0.01, ***P < 0.001.

As the recording of somatic calcium signals is widely used for the monitoring of neuronal functional activity *in vitro*, with spiking activity in neurons inferred from somatic calcium signals, we sought to evaluate whether differences in calcium activity were evident during the latter period of the differentiation protocol (Days 60–100) (Supplementary Fig. 4A and B). A significantly higher level of overall calcium activity was noted in each of the *SGCE*-mutation-positive lines, compared to isogenic controls, at time points Days 60, 80 and 100 [Fig. 3A(i)–(iii)]. However, within these active cells those lines with *SGCE* mutations demonstrated a significantly fewer calcium transients and longer interspike intervals (Supplementary Fig. 4C and D), as well as changes to the calcium event shape that included: smaller amplitudes, longer rise times and shorter fall times (Supplementary Fig. 4E–G), compared to wild-type controls, indicating that, although the *SGCE*-mutation-positive cell lines demonstrated a higher overall level of activity, these events were smaller and took longer to peak than their wild-type counterparts. In addition, individual application of the L-type calcium blocker, nifedipine, competitive AMPA antagonist (cyanquixaline, CNQX) and selective NMDA antagonist (AP5) resulted in marked reduction of the calcium responses across all cell lines (Supplementary Fig. 4H–J). While calcium imaging provides some indication of collective functional activity, MEAs were also used to better understand network-level neuronal activity. Here, significantly higher numbers of spikes, bursts and network bursts were observed across all *SGCE* mutation lines, compared to their isogenic controls [Fig. 3B(i–iii)], together with significantly higher SPIKE values [Fig. 3B(iv)], providing a measure of spike train synchrony.^35^ These differences are also visible in the raster plots from lines Pt1 and Pt1C, provided as an example for comparison [Fig. 3C(i–iv)]. Collectively, the Ca^2+^ imaging and MEA approaches indicate that loss of ε-sarcoglycan expression in cortical neurons increases functional network activity, alongside changes to individual events patterns. We next sought to determine the impact of these differences at a single-cell level through whole-cell patch clamp measurements in current-clamp mode at Days 60 and 80, categorizing attempts at action potential (AP) generation into no AP, attempted AP, AP, attempted AP train and AP train. Across all paired cell lines, we found that absence of e-sarcoglycan expression increased the propensity for AP generation, with higher proportions of APs, attempted AP trains and AP trains [Fig. 4A(i–ii)] at both time points and increasing as anticipated between Days 60 and 80. An example of the traces captured of evoked activity from current injection steps from individual cell lines are provided in Fig. 4B(i) *SGCE*wt, (ii) SGCEko, (iii) Pt1C and (iv) Pt1). Analysis of individual AP characteristics found no significant differences (*P* > 0.05) between *SGCE*-mutation-positive and wild-type pairs for any of the neuronal baseline membrane properties, including resting membrane potential (RMP), input resistance, membrane time constant and capacitance (Supplementary Fig. 5). By contrast, significant differences were seen across multiple AP characteristics, including larger amplitudes (*P* < 0.001–0.03) and shorter AP half-widths (*P* < 0.001–0.02, except for Pt3, *P* = 0.2), faster AP rise and fall times (*P* < 0.001–0.03) (Fig. 4C) and more negative AP threshold values (*P* < 0.001–0.03) [Supplementary Fig. 5E(i–iv)] in lines carrying an *SGCE* mutation, providing further insight of the changes underpinning the observed increased activity in these lines.

### Gene expression analysis suggests differences in neuronal morphology and synaptic function may contribute to the observed functional changes

**Figure 5:**
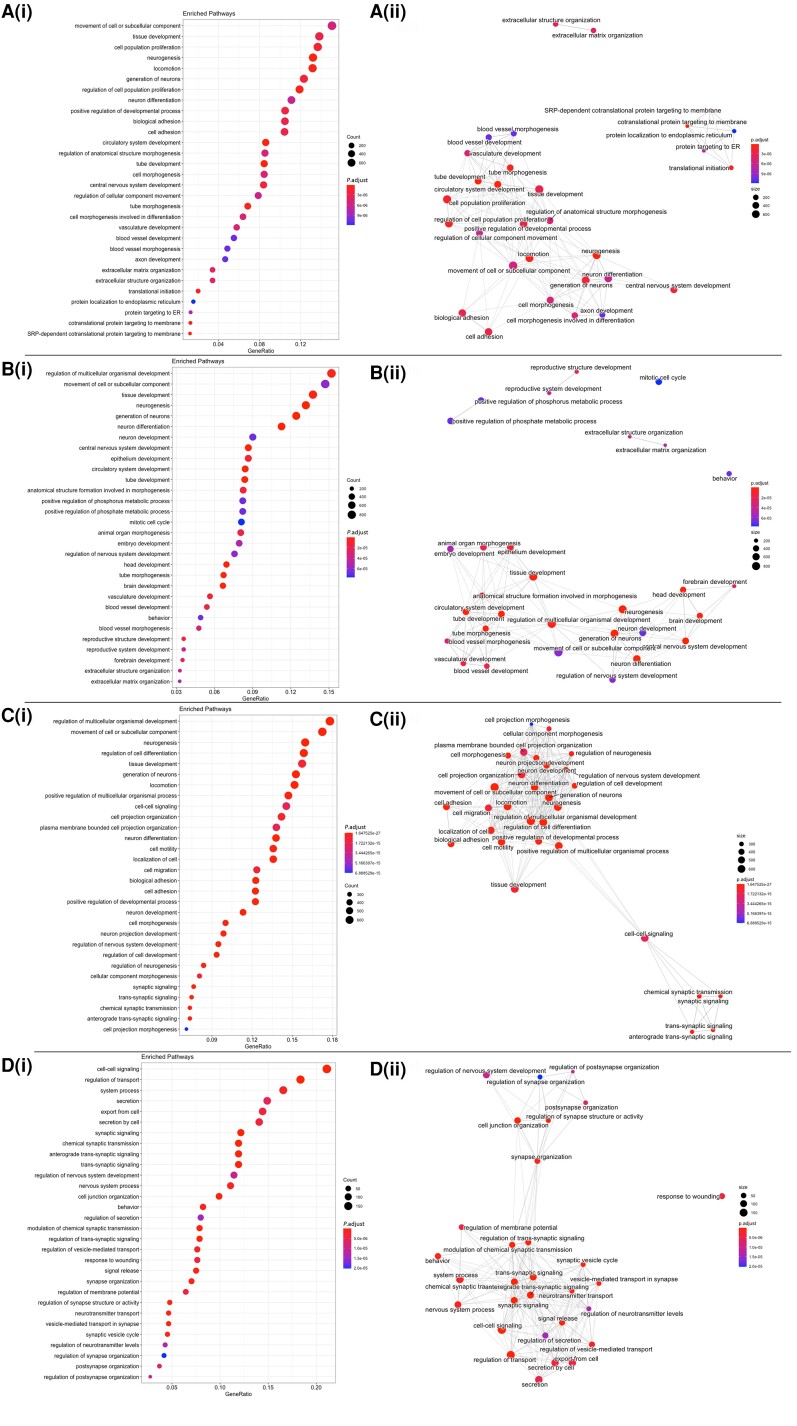
**Bulk RNA-seq analysis comparing *SGCE*wt and *SGCE*ko hESC lines at Days 60, 80 and 100.** Dot plot representation of the top overexpressed 30 GO terms in *SGCE*ko line compared to *SGCE*wt at Day 60 [**A**(**i**)], Day 80 [**B**(**i**)], Day 100 [**C**(**i**)], and comparison of *SGCE*wt versus *SGCE*ko between Days 80 and 100 [**D**(**i**)]. GO terms are sorted by their adjusted *P*-value with Banjamini-Hochberg *P*-value correction of FDR < 0.05. GO term enrichment analysis of the top 30 categories presented as enrichment maps for *SGCE*ko: *SGCE*wt comparison at Day 60 [**A**(**ii**)] , Day 80 [**B**(**ii**)] , Day 100 [**C**(**ii**)], and *SGCE*wt versus *SGCE*ko changed between Days 80 and 100 comparison [**D**(**ii**)]. GO functional groups exhibiting higher statistically significant differences using Benjamini–Hochberg *P*-value correction (FDR < 0.05) are shown. Network graph nodes represent GO terms (the most significant are named), and edges indicate shared genes between GO terms. Within this the nodes represent gene-sets, and the edges represent mutual overlap, such that highly redundant gene sets are grouped together as clusters, enabling visual interpretation of these relationships.

To investigate the neurodevelopmental effects of the absence of ε-sarcoglycan expression, we undertook bulk RNA sequencing for analysis of DEGs between the *SGCE*wt and *SGCE*ko hESC lines at Days 60, 80 and 100 of cortical neuronal differentiation (Supplementary Fig. 6A). Comparison of the *SGCE*ko to *SGCE*wt lines, following constraint for protein-coding genes and FDR correction at each time point, revealed 5689 DEGs at Day 60 (2841 upregulated, 2848 downregulated), 6218 DEGs at Day 80 (2878 upregulated, 3340 downregulated) and 4260 DEGs at Day 100 (1801 upregulated, 2459 downregulated) (Supplementary Tables 1–3 and Supplementary Fig. 6B–D). GO analysis of the protein coding DEGs revealed a strong enrichment for genes involved in neuronal development (‘neurogenesis’, ‘neuron differentiation’, ‘neuron development’) across all three time points [Figs 5A(i), 3B(i) and 3C(i)]. Other enriched ontological pathways included terms relating to neuronal morphological development such as ‘axon development’ (Day 60) (adjusted *P*-value = 1.07 × 10^−05^) and ‘neuron projection development’ (Day 100) (adjusted *P*-value = 2.31 × 10^−19^), with the degree of enrichment of these pathways increasing over this interval of differentiation. More evident towards the latter stages of differentiation was enrichment for components of synaptic function, including ‘synaptic signalling’ and ‘synaptic transmission’ (Day 100), providing further evidence of the ongoing *in vitro* neuronal maturation of these lines. Considering the widening difference in functional activity between the *SGCE*-mutation-positive and wild-type lines in the later stages of the differentiation protocol, we examined for differences in the change in expression between *SGCE*wt and *SGCE*ko lines between Days 80 and 100 through an interaction analysis [Fig. 5D(i)]. In addition to the pathways identified for neuronal morphology, synaptic pathways were further enriched, notably those linked with synaptic signalling and neurotransmission (‘synaptic signalling’, ‘regulation of trans-synaptic signalling’, ‘regulation of synaptic structure or activity’).

Gene set enrichment maps were generated for comparisons at each time point to gain greater understanding of how these pathways interact, and their changing relationship over time. DEGs from *SGCE*ko versus *SGCE*wt contrasts at Day 60 were characterized by a predominant network of neuronal development pathways, alongside a smaller cluster relating to protein handling by the endoplasmic reticulum, likely of importance given previous evidence for a role of the endoplasmic reticulum in the handling of misfolded mutant ε-sarcoglycan proteins in the context of *SGCE* mutations [Fig. 5A(ii)].^10^ Enrichment maps at Days 80 and 100 demonstrated more densely packed neurodevelopmental networks [Fig. 5B(ii) and C(ii)], while synaptic pathways formed an emerging distinct cluster at Day 100, linked by pathways involved in cell-cell signalling. Synaptic pathways formed the only cluster when comparing differences between *SGCE*wt and *SGCE*ko between Days 80 and 100 [Fig. 5D(ii)], segregating into two subgroups, with one more focused on synaptic organization, and the other, synaptic activity. Overall, these results support a progressive maturation of the neurons during differentiation but suggest that the functional differences observed above could be attributable to differences in neuronal morphological development and changes to synaptic structure and function, pathways highlighted to be of importance across other forms of inherited dystonia.^36^

Independent enrichment analysis of the upregulated (Supplementary Fig. 7) and downregulated (Supplementary Fig. 8) genes in the *SGCE*ko lines compared to their *SGCE*wt matched controls found ‘neuron differentiation’, ‘neuron development’, ‘axonogenesis’ and ‘trans-synaptic signalling’ in the upregulated gene set, with further evidence of distinct segregation of neuronal morphology and synaptic pathways, increasing at subsequent time points (Supplementary Fig. 7) In contrast, enrichment analysis of the downregulated genes found ‘protein transport’, ‘cell adhesion’, ‘cell migration and extracellular structure organization’ to be consistently enriched, although the segregation of protein handling and extracellular organization pathways into distinct clusters was less distinct than that observed between the two main clusters in the upregulated pathways (Supplementary Fig. 8).

### *SGCE* mutations are associated with a more complex dendritic arbor and longer axon initial segment

**Figure 6:**
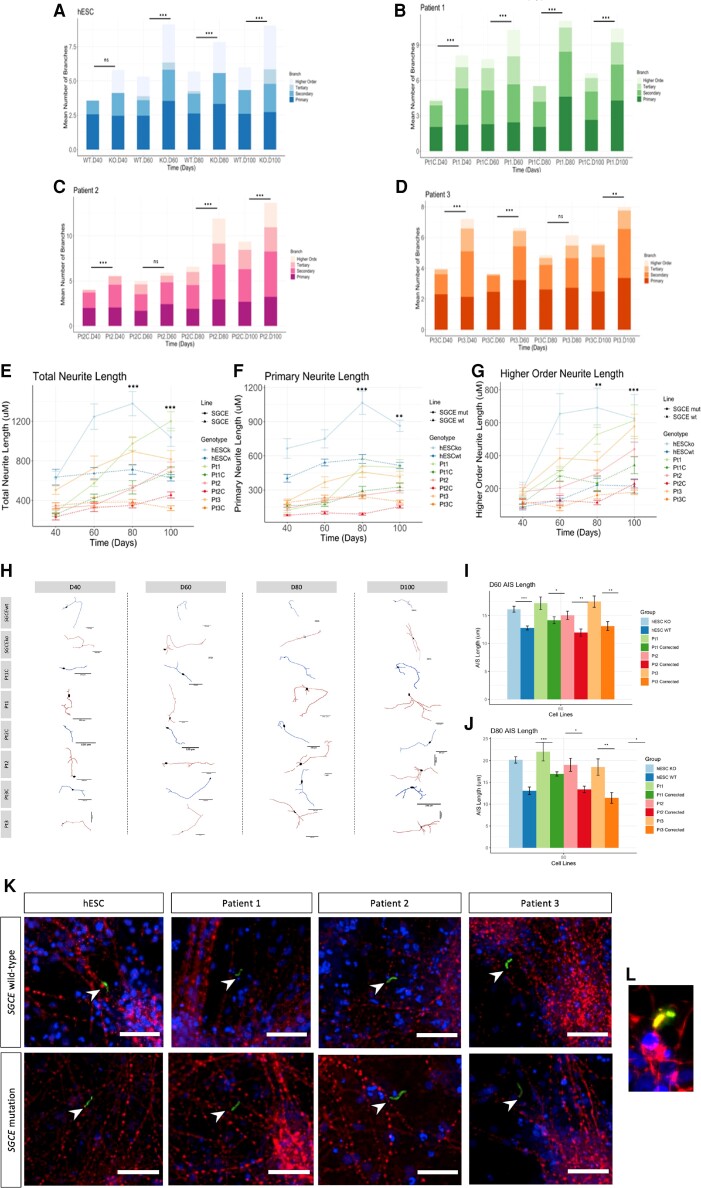
**Investigation of the development and complexity of the dendritic arbor**. (**A**–**D**) Stacked bar graphs demonstrating the number of primary, secondary, tertiary and higher order (beyond tertiary) branches evident at Days 40, 60, 80 and 100 of differentiation. Isogenic paired lines (*SGCE* mutant and wild-type control) compared; hESC (**A**), Pt1 (**B**), Pt2 (**C**), Pt3 (**D**). Statistical comparison with two-way ANOVA at each time point. ns = not significant, *P < 0.05, **P < 0.01, ***P < 0.001. (**E**–**G**) Line plots depicting the change in neurite length over time (Days 40–100), total neurite length (**E**), primary neurite length (**F**) and higher order (all neurites beyond primary neurites) neurite length. Data are presented as mean ± SEM from three independent experiments per line. Statistical comparison over time using two-way ANOVA analysis. ns = not significant, *P < 0.05, **P < 0.01, ***P < 0.001. (**H**) Representative neurite traces of each cell line across Days 40, 60, 80 and 100 of differentiation. Scale bars = 100 μm. (**I** and **J**) Quantification of AIS length at Days 60 (**I**) and 80 (**J**). Data are presented as mean ± SEM from three independent experiments per line. Statistical comparison between each line pair (*SGCE* mutant and wild-type) using one-way ANOVA at each time point (Days 60 and 80). ns = not significant, *P < 0.05, **P < 0.01, ***P < 0.001. (**K**) Representative immunofluorescence of the AIS (green) alongside nuclear staining with DAPI (blue) and neuronal marker, MAP2 (red). Scale bars = 100 μm. (**L**) Magnified representative image of the AIS (green) in relation to the nucleus (blue) and neurites (red).

In light of the hESC (*SGCE*wt and *SGCE*ko) bulk-RNAseq analysis, we first sought to examine for differences in neuronal morphology given its potential for influence on functional activity, although previous studies have suggested that the structure of the dendritic arbor may also be influenced by the electrophysiological properties of the cell.^37,38^ Using sparse labelling of neurons with a GFP expression vector (Supplementary Fig. 9A), we examined dendritic and axonal projections at 20-day intervals between Days 40 and 100, initially dividing the branch types into primary, secondary tertiary and higher order (all branches beyond tertiary branches). Here, *SGCE*-mutation-positive lines had a significantly higher number of branches, most notably secondary, tertiary and higher order branch types, at almost all time points, but consistently evident at Day 100 (Fig. 6A–D). We next sought to examine whether any changes in neurite length were evident, examining total neurite length, primary branch length and higher order length (all branch types beyond primary branches). Considering significant only those time points in which a significant statistical difference was observed between the *SGCE*-mutation-positive and wild-type cell lines for each of the line pairs, we observed significantly longer total neurite length, primary and higher order branches at both Days 80 and 100 (Fig. 6E–G), ultimately resulting in a more complex dendritic arbor (Fig. 6H). No significant differences were observed at Day 40, and although some significantly longer branches were seen in the *SGCE*-mutation-positive lines at Day 60 (hESC: total neurite length, Pt2: primary branches, Pt3: higher order branches), these were not consistently demonstrated across all lines. We next explored evidence of structural changes to the axon initial segment (AIS), an axonal region dense in voltage-gated ion channels and whose length and position are recognized as contributing to AP generation and overall neuronal excitability.^39–41^ Ankyrin G immunofluorescence, widely used as a marker for the AIS, at Days 60 and 80 of differentiation (Fig. 6K) found this segment to increase in length between these two time points, as well as to be significantly longer in those cell lines harbouring an *SGCE* mutation, compared to their wild-type control (Fig. 6I and J).

### Disruption to synaptic adhesion molecules neurexin-1 and neuroligin-4 levels identified in *SGCE* mutation carrying lines

**Figure 7:**
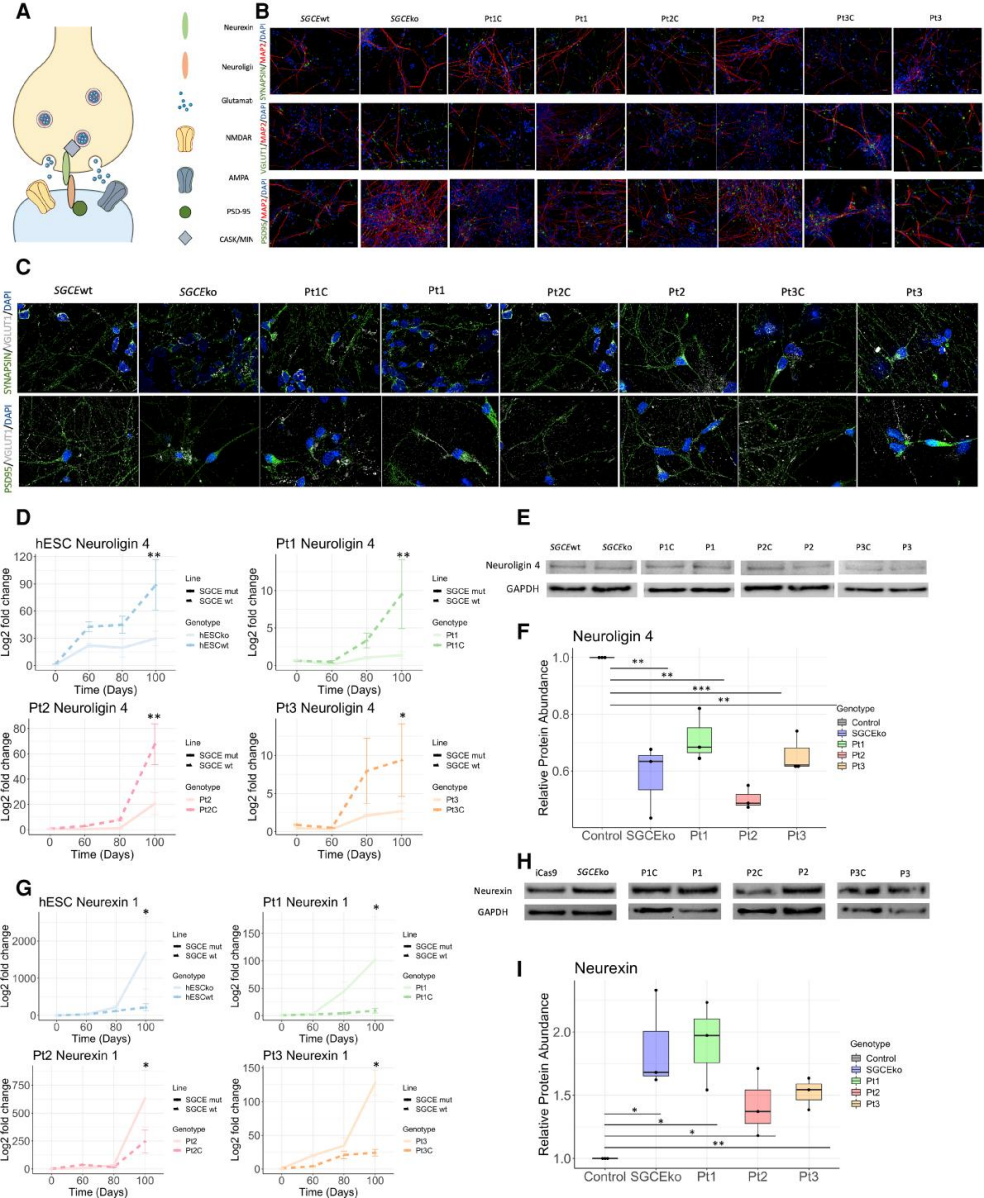
**Examination of the impact of loss of ε-sarcoglycan expression on synaptic components**. (**A**) Schematic illustration of cortical excitatory glutamatergic synapse. (**B**) Representative immunofluorescence images for synaptic proteins—synapsin, VGLUT1 and PSD95—together with neuronal marker, MAP2 and nuclear marker, DAPI. Representative images shown for all four isogenic control and *SGCE* mutation carrying lines at ×40 magnification and Day 80 differentiation. (**C**) Representative immunofluorescence images for synaptic proteins, synapsin, VGLUT1 and PSD95 and nuclear marker, DAPI. Representative images shown for all four isogenic control and *SGCE* mutation carrying lines at ×100 magnification and Day 80 differentiation. (**D**) qRT-PCR for neuroligin-4 expression at Days 0, 60, 80 and 100. Each *SGCE* mutation carrying line compared to their wild-type isogenic control. Data are presented as mean ± SEM from three independent experiments per line. Lines compared using two-way ANOVA analysis, with *P < 0.05, **P < 0.01, ***P < 0.001. (**E**) Representative immunoblot for neuroligin-4 and loading control (GAPDH). (**F**) Quantification of relative neuroligin-4 abundance in total neuronal cell lysates. (*n* = 3 for all). Data are presented as mean ± SEM. All data compared to the isogenic control cell lines for each *SGCE* mutation carrying lines. This is presented as a single control point (*n* = 1) for ease of comparison. Statistical comparison using one-way ANOVA analysis. *P < 0.05, **P < 0.01, ***P < 0.001. (**G**) Quantitative RT-PCR for neuroligin-4 expression at Days 0, 60, 80 and 100. Each *SGCE* mutation carrying line compared to their wild-type isogenic control. Data are presented as mean ± SEM from three independent experiments per line. Lines compared using two-way ANOVA analysis, with *P < 0.05, **P < 0.01, ***P < 0.001. (**H**) Representative immunoblot for neurexin-1 and loading control (GAPDH). (**I**) Quantification of relative neurexin-1 abundance in total neuronal cell lysates (*n* = 3 for all). Data are presented as mean ± SEM. All data compared to the isogenic control cell lines for each *SGCE* mutation carrying lines. This is presented as a single control point (*n* = 1) for ease of comparison. Statistical comparison using one-way ANOVA analysis. *P < 0.05, **P < 0.01, ***P < 0.001.

Gene expression analysis also identified synaptic structure and function as a key difference between *SGCE*wt and *SGCE*ko lines, also implicated in other genetic forms of dystonia, and other neurological and psychiatric disorders.^36,42^ A simplified schematic illustration of a cortical excitatory glutamatergic synapse (Fig. 7A) outlines the multiple components that contribute to its function, with these including synaptic proteins (synapsin, PSD95, VGLUT1), receptors (AMPA, NMDA) and adhesion molecules (neurexin, neuroligin). RT-qPCR and western blot quantification of multiple key synaptic proteins; synaptophysin (Supplementary Fig. 9B), synapsin (Fig. 7B and C and Supplementary Fig. 9J and K), PSD95 (Fig. 7B and C and Supplementary Fig. 9C, L and M) and VGLUT1 (Fig. 7B and C and Supplementary Fig. 9N and O) identified no significant differences in gene or protein expression associated with ε-sarcoglycan expression (*P* > 0.05). Gene expression levels also gradually increased during differentiation, in keeping with progressive synaptic maturation. Synaptic receptors, namely NMDA and α-amino-3-hydroxy-5-methyl-4-isoxazolepropionic acid receptors (AMPA) receptors, are also key contributors of cortical glutamatergic synapses, with involvement in multiple processes including synaptic plasticity and calcium signalling.^43,44^ Gene expression quantification of their multiple subunits again demonstrated the anticipated increase in levels over the course of the differentiation protocol, but no significant differences between *SGCE*-mutation positive cells and their wild-type controls (Supplementary Fig. 9D–I). Of the multiple known synaptic adhesion molecules, neurexins and neuroligins are perhaps the most extensively described, acting as calcium-dependent cell adhesion molecules involved in the formation of both excitatory and inhibitory synapses *in vitro*, with changes in expression also shown to impact dendritic morphology.^45–47^ Neuroligin-4 was of particular interest given its predominant brain expression, most notably within the postsynaptic membrane of cortical glutamatergic synapses, with previous studies having demonstrated its ability to modulate excitatory synaptic transmission.^48,49^ Here, significant differences were observed when examining the presynaptic neurexin-1, and postsynaptic neuroligin-4 molecules with quantification of protein (Day 80) and gene expression (Day 100) identifying significantly lower neuroligin-4 (*P* < 0.01) and significantly higher neurexin-1 (*P* < 0.05) levels in *SGCE*-mutation-positive cell lines, compared to controls (Fig. 7D–I).

## Discussion

Using two distinct approaches, hESC CRISPR gene-edited cell lines and patient-derived iPSC, this study generated four cell line pairs, each with an *SGCE* mutation and isogenic wild-type control, to examine potential cortical neuronal changes that may account for the clinical phenotype observed in myoclonus dystonia. Consistent findings across all cell line pairs identified no differences in the early expression of cortical neurodevelopmental markers. By contrast, functional analyses identified increased neuronal activity across all *SGCE*-mutation-positive lines, manifesting in higher numbers of AP spikes, bursts and network bursts, together with an earlier and greater propensity towards AP firing on an individual cell level. Calcium imaging also demonstrated an overall increase in calcium activity in the *SGCE*-mutation-positive lines compared to controls, in conjunction fewer calcium transients and increased interspike interval suggesting additional disruption to Ca^2+^ handling. Gene expression analysis of the hESC *SGCE*wt and *SGCE*ko lines, aimed at exploring potential mechanisms contributing to this increased functional activity, identified changes to axonal morphology and synaptic signalling. Subsequent experiments identified *SGCE*-mutation-positive cell lines to have a more complex neurite structure and longer voltage-gated channel rich AIS. Synaptic analysis found disruption to adhesion molecule expression, namely increased presynaptic neurexin-1 and decreased postsynaptic neuroligin-4 levels in association with *SGCE* mutations.

The consistency in expression of cortical developmental and neuronal markers across *SGCE* mutation and control cells lines is consistent with previous patient-derived myoclonus dystonia iPSC cortical neuronal models, and in keeping with the absence of gross structural abnormalities on MRI brain imaging of these patients.^34,50^ It should be noted that some degree of variability in the differentiation efficiency is observed between the lines, most notably the patient-derived lines (Fig. 2D–F). However, a key element of this study is the comparison of each *SGCE* mutation carrying line with an isogenic matched control line harbouring the wild-type sequence. Here, each of the cell line pairs demonstrate a similar degree of differentiation efficiency variability, while the patient wild-type control lines (Pt1C, Pt2C and Pt3C) demonstrate greater variance between subjects, further reinforcing the benefits of this approach. In line with this previous work, we have demonstrated preservation of the maternal imprinting recognized to influence inheritance of pathogenic *SGCE* mutations throughout the reprogramming and differentiation process, making this a representative model for myoclonus dystonia. We also demonstrated the absence of off-target effects through both SNP-based CNV array (changes >100 000 bp in size) and direct sequencing of the target *SGCE* exon, used to identify smaller off-target variants within the active gene editing window. This approach, however, does not account for potential smaller (<100 000 bp) off-target variants elsewhere in the genome that may impact the observed phenotype. However, the phenotypic analyses have demonstrated consistent findings across all four of the cell-line pairs, with likelihood of the same off target effect being introduced, across both iPSC and hESC lines, unlikely. The number and morphology of neural rosettes, features thought to recapitulate the cellular organization of the neural tube, also demonstrated no significant differences between the cell lines, indicating that any impact cortical neuronal development likely involves later stages of development.^51–53^

As discussed above, increasing evidence supports dystonia being a network-based disorder involving basal ganglia-cerebello-thalamo-cortical circuits, influenced by the balance of excitatory and inhibitory activity. Although human *in vivo* electrophysiological studies have suggested that the primary cause of the observed hyperexcitability is loss of inhibition,^54,55^ others have shown that this cannot be accounted for by loss of inhibition alone. This study further supports this, suggesting increased intrinsic cortical neuronal excitability with a hyperexcitable phenotype observed across both network (MEA and calcium imaging), and individual cell measurements (single-cell patch clamp), consistent with other dystonia models.^56^ Calcium imaging analysis found *SGCE*-mutation-positive lines to exhibit a higher percentage of calcium activity, fewer calcium transients and longer interspike interval, compared to wild-type controls. Cytosolic calcium measurements are determined by cellular influx via voltage-gated calcium channels and efflux from intracellular stores, principally the endoplasmic reticulum.^57^ In addition, application of the L-type calcium channel blocker, nifedipine, in this study resulted in loss of the calcium signal across both the *SGCE*-mutation-positive and wild-type controls, suggesting that influx of extracellular calcium is key in generating the calcium transients observed although the differences observed between wild-type and mutation carrying lines may also be attributable to the distinct handling of calcium from intracellular stores. Disruption to calcium signalling has been identified in disease models of other genes implicated in myoclonus dystonia, including *CACNA1B* linked with changes to N-type calcium channel function,^58,59^ disruption to endoplasmic reticulum calcium release in *KCTD17* mutation models and *ANO3* dystonia.^60,61^ In addition, a recent SGCE-mutation-positive patient-derived iPSC model, differentiated towards a medium spiny neuron (MSN) lineage also demonstrated significantly higher basal intracellular Ca^2+^ levels and smaller transient amplitudes, although found no significant difference in the percentage of spontaneous Ca^2+^ signals. These results potentially indicate that disrupted calcium handling, particularly of intracellular stores, represents a common theme in dystonia pathogenesis, although these changes may differ between neuronal types.^61,62^

Evaluation of the differences in single-cell AP shape provide further insights into the potential mechanisms underlying the observed hyperexcitability. Here, *SGCE* mutations were associated with more negative AP thresholds, larger amplitudes, shorter half-widths, and faster rise and fall times. Larger AP amplitudes and shorter half-widths are both considered markers of functional maturity, in part due to increasing numbers of functional cell membrane ion channels. Our results suggest a dysregulation of this process, indicating that ε-sarcoglycan may have a role in constraining neuronal activity during development. It should be noted however, that the resting membrane potential observed for each of our lines (approximately −20 mV) is somewhat higher than that observed in other studies using the same protocol (approximately −40 mV) (Supplementary Fig. 5A). While this potentially indicates a lower level of cell maturity, these results were remarkably consistent across all of the cell lines, and across multiple differentiations. In addition, the MEA analysis demonstrated evidence of spontaneous APs, suggesting sufficient cellular maturity to demonstrate this level of functional activity. There have been few *in vitro* electrophysiological studies of dystonia, however one such rat model of *DYT12* dystonia found cerebellar Purkinje cells to show erratic burst firing secondary to sodium pump dysfunction.^63^ A single-cell RNAseq study of iPSC-derived neurons also found strong correlation between action potentials, synaptic activity, dendritic complexity and gene expression,^64^ suggesting that those neurons with the most mature action potential properties also have the highest expression levels of voltage-gated Na^+^ and K^+^ channels. K_v_7 potassium channel upregulation has also been identified in striatal neuronal development and thought to promote maturation of human iPSC-derived neurons,^65^ while pathogenic gain-of-function K_v_1.2 channel mutations have been linked with an hyperexcitable epilepsy phenotype.^66^

The increased AIS length in the *SGCE*-mutation-positive lines provides another potential explanation for the observed increased functional activity. The AIS not only represents a specialized membrane region from which APs are generated, it also demonstrates activity dependent structural and functional plasticity, and receives exclusive synaptic input from inhibitory GABAergic interneurons, neuronal subtypes already implicated in dystonia pathophysiology.^39,67^ Key to this role is the high density clustering of voltage-gated ion channels within the region, expressed in compartmentalized regions dependent on their roles.^40^ Predominant channels include, Na+ (Na_v_1.1, 1.2, 1.6) and K+ (K_v_1, 2.2) channels with the Na_v_1.6 channel clustered in greatest density proximally, allowing for the main inward rapid depolarizing current, as well as lowering the current threshold required to trigger an AP.^68^ The other Na channels (Na_v_1.1 and Na_v_1.2), both thought to contribute to regulating the back propagation of APs to the soma, and have been linked to pathophysiological roles in epilepsy and autism, while Na_v_1.6 channel dysfunction has been identified in models of schizophrenia, depression and autism.^69,70^ More recently, stem cell models of the paroxysmal dyskinetic disorder caused by *PRRT2* (DYT8) mutations also identified a hyperexcitable cortical neuronal phenotype, demonstrating a longer AIS with PRRT2 acting as a negative regulator of Na_v_1.2 and Na_v_1.6 channels.^71^ The distribution of K-channels also varies throughout the AIS, with K_v_1.1 and K_v_1.2 predominantly found in the more distal portion, as well as appearing later in development compared to their sodium counterparts.^72,73^ The AIS also demonstrates considerable functional plasticity through variation in both its position and length. A recent study sought to model the impact of neuronal morphology on this excitability, finding that neurons with larger dendritic trees, such as cortical pyramidal neurons, were most excitable when the AIS was longer and/or located further from the soma.^74^ Collectively, this suggests that changes to ion channel expression and function within the AIS may contribute to the increased excitability observed within the *SGCE*-mutation-positive lines. Further work will be needed to better characterize these components and seek to determine whether they represent potential future therapeutic targets.

Changes to neurite morphology, as observed in the ε-sarcoglycan deficient lines, are influenced by multiple factors, including neuronal activity, cell surface receptors, adhesion molecules, and regulators of the actin cytoskeleton,^75–77^ with neurite structural abnormalities recognized as potential contributors to multiple neurodevelopmental disorders.^78,79^ Calcium-signalling is also integral in regulating the formation and plasticity of dendritic branching, with postsynaptic rises in calcium levels essential for activity-dependent plasticity.^38,80,81^ Gene expression studies in *Thap1* (DYT6) murine models have identified neurite development as an enriched pathway across multiple brain regions, with signalling by the Rho family of GTPases, regulators of the actin cytoskeleton, being a key pathway.^82,83^ However, the relationship between greater neurite length, and neuronal and calcium activity observed in this study, may be bidirectional, with multiple studies also showing that changes to dendritic complexity can influence neuronal firing patterns and action potential kinetics.^84,85^ Most notably, LTP—a clinical electrophysiological feature consistently observed across dystonia cohorts—has also been shown to increase the number of dendritic spines with associated increases in synaptic strength.^86^

Investigation of synaptic structure found no significant differences in the number of synapses formed, reflected in synapsin and PSD-95 levels, but significantly higher levels of presynaptic neurexin-1 and lower levels of postsynaptic neuroligin-4 in the *SGCE*-mutation-positive lines compared to their isogenic controls. Neurexins and neuroligins form one of multiple pairs of adhesion molecules at neuronal synapses, providing not only a mechanical link but also participating in synapse formation and dendritic and synaptic morphological regulation.^87^ They also modulate presynaptic calcium channel function, with loss of alpha-neurexin resulting in decreased whole cell calcium currents but no changes in N-P/Q-type calcium ion channel expression.^88^ Neurexins also bind to dystroglycan in a calcium-dependent manner, with murine dystroglycan knock out models demonstrating impaired synaptic electrophysiological properties.^89^ Four subtypes of Neuroligin are now recognized with murine studies demonstrating that loss of neuroligin expression impacts synaptic maturation and neurotransmission, but with no effect on the number of synapses formed, in keeping with the results observed here.^90^ Neuroligin-4 is of particular interest as *NLGN4* gene mutations have been reported in patients with autism and other neurodevelopmental disorders. Similar to that previously reported with missense *SGCE* mutations,^10^ missense *NLGN4* mutations also show impaired N-linked glycosylation, intracellular Golgi and endoplasmic reticulum retention and reduced surface trafficking of the NLGN4 protein. Through a combination of overexpression and knock-out analyses, reduction in postsynaptic NLGN4 expression is believed to increase synaptic excitation through prevention of the wild-type NLGN4 function in silencing excitatory synapses, providing a mechanism for further exploration in the context of *SGCE* mutations.^91^

Although iPSC lines were derived from only three unrelated patients in this study, two of whom carried the same missense mutation (Patients 2 and 3) potential links between clinical and cellular phenotypes have emerged. A more detailed summary of the clinical phenotypes for Patients 1 and 2 have been reported previously.^9^ In brief, Patient 1 demonstrates a more severe motor and non-motor phenotype, substantially impacting both daily living and quality of life. Patient 1 has subsequently undergone bilateral globus pallidus internus (GPi) deep brain stimulation (DBS) resulting in near amelioration of their motor symptoms. Motor and psychiatric phenotypes in Patients 2 and 3 are milder, managed with no intervention or oral medical therapy respectively. Interestingly, Patient 1 also consistently demonstrated higher MEA-recorded network activity as well as longer neurite and AIS lengths. Whether these differences directly relate to clinical phenotype is speculative at present, but represents an interesting area for future evaluation, requiring multiple cell lines carrying distinct mutations.

In summary, this study demonstrates an increase in cortical glutamatergic neuronal activity in the context of *SGCE* mutations, known to result in the hyperkinetic movement disorder, myoclonus dystonia. Alongside these functional changes, development of a more complex neurite morphology and disruption to synaptic adhesion molecules are also observed. Future work will involve more detailed determination of the relative contribution of these components to the resultant phenotype, whether the primary driver to these changes can be identified, and whether one or multiple of these components have the potential to be harnessed as targets for future therapeutic drug development.

## Supplementary Material

awac365_Supplementary_Data
