# Supplementary material for: Cortical neuronal hyperexcitability and synaptic changes in SGCE mutation-positive myoclonus dystonia
Source: Brain. 2022 Oct 7;146(4):1523–41. doi: 10.1093/brain/awac365 (PMC10115238; doi:10.1093/brain/awac365)
Supplement: awac365_Supplementary_Data [file awac365_supplementary_data.zip › brain-2022-00185-File010.pdf]

Supplementary Figures and Methods Tables

Supplementary Figure 1 (Figure S1)

A

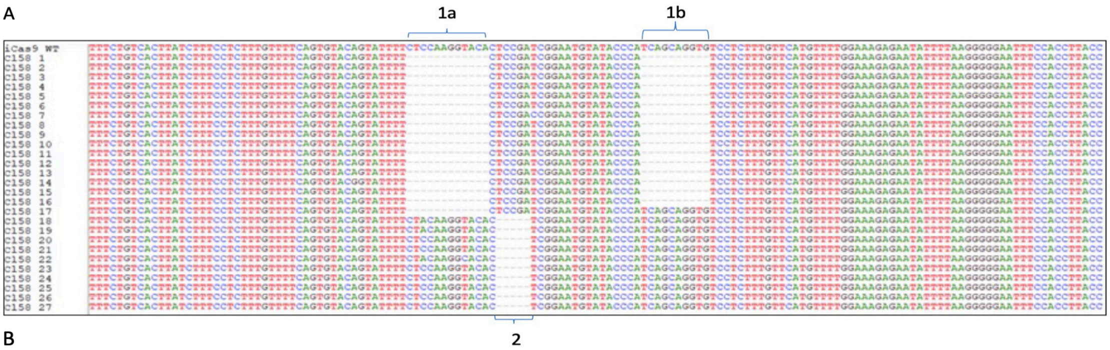

B

Clone 58	Allele	Type of sequence variant	Sequence (insertion/deletion)	Position (relative to transcription start site)		Genotype	Protein coding change	Predicted pathogenicity
	1a	Deletion	CTCCAAGGTACA	123	134	c.127_138 del	p.lys43_ser46del	Provean score <-2.5: deleterious
	1b	Deletion	TCAGCAGGTG	157	166	c.270_279 del	p.ala54serfs*29	Provean score <-2.5: deleterious
	2	Deletion	TCCGA	136	140	c.138_142 del	p.asp47glufs*20	Provean score <-2.5: deleterious

C(i)

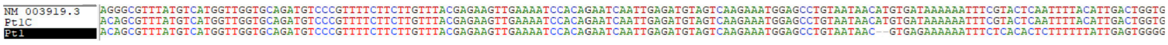

C(ii)

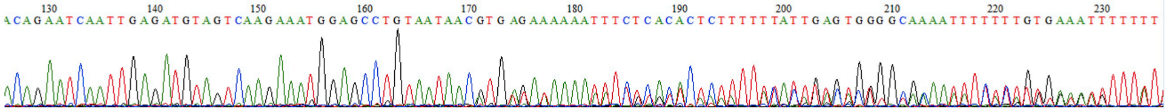

C(iii)

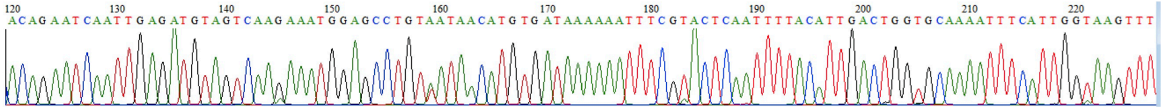

C(iii)

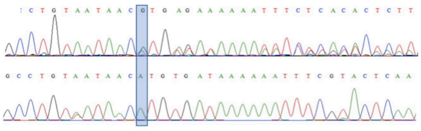

D (i)

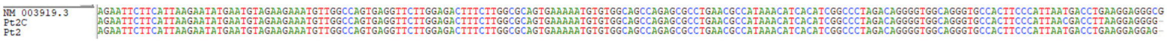

D (ii)

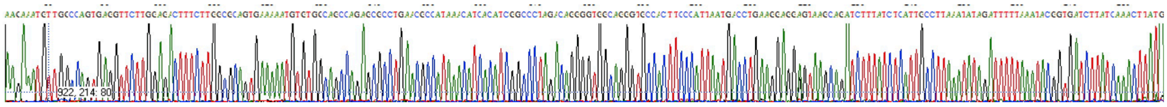

D (iii)

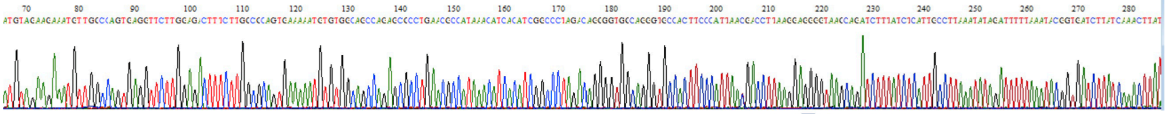

D (iv)

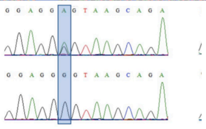

E (i)

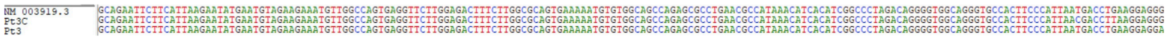

E (ii)

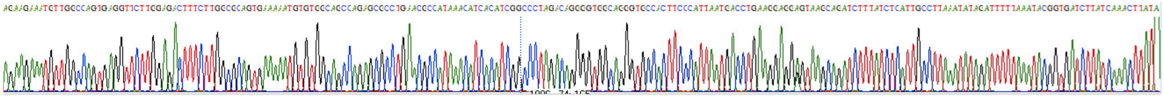

E (iii)

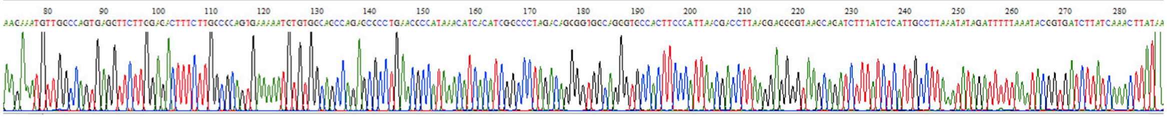

E (iv)

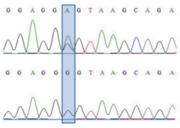

Figure S1: A: Direct sequencing results for *SGCE*wt control (n=3 clonal lines) and *SGCEko* (n=27 clonal lines) demonstrating the exon 2 compound heterozygous mutation resulting from CRISPR/Cas9 gene editing. **B:** Description of the identified *SGCE*ko compound heterozygous mutation, and the *in silico* predictive effect of each deletion on the translated epsilon-sarcoglycan protein. All were anticipated to have a deleterious impact on the structure and function of the resultant protein. **C-E:** DNA direct sequencing demonstrated the pathogenic *SGCE* mutations in each of the Patient lines. These include each of the paired patient lines (*SGCE*-mutation positive and corrected control) against the *SGCE* reference sequence NM_003919.3, (i) Sanger sequencing chromatogram for *SGCE* mutation positive (ii) and corrected (iii) lines, and magnified image to demonstrate the mutation in greater detail (iv). Sequencing includes the nonsense mutation in Pt1 (C), and missense mutations in Pt2 (D) and Pt3 (E).

Supplementary Figure 2 (Figure S2)

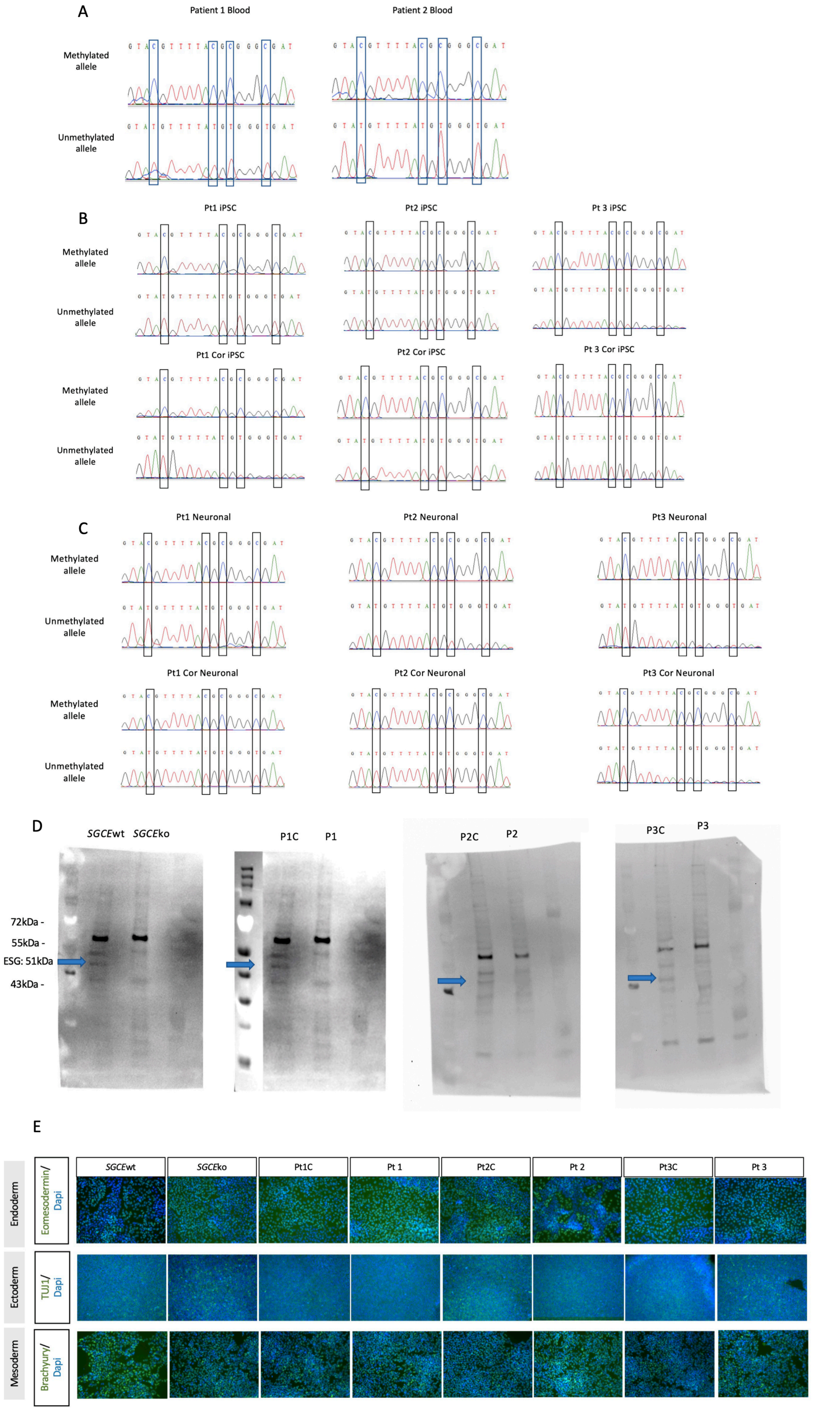

<u>Figure S2:</u> Direct sequencing traces demonstrating the preservation of imprinting in the patient derived lines following blood sample collection (**A**), cellular reprogramming to a pluripotent state (**B**) and differentiation towards a neuronal lineage (**C**). **D:** Full length western blot for Figure 1D: Representative immunoblot for Epsilon-sarcoglycan and loading control (GAPDH), including *SGCE*wt and *SGCE*ko, Pt1C (Patient 1 wild-type control), Pt1 (Patient 1), Pt2C (Patient 2 wild-type control), Pt2 (Patient 2), Pt3C (Patient 3 wild-type control), Pt3 (Patient 3).**E:** Immunofluorescence representative images for Eomesodermin (endoderm), TUJ1 (ectoderm) and Brachyury (mesoderm) expression from spontaneous *in vitro* differentiation of all 8 cell lines.

Supplementary Figure 3 (Figure S3)

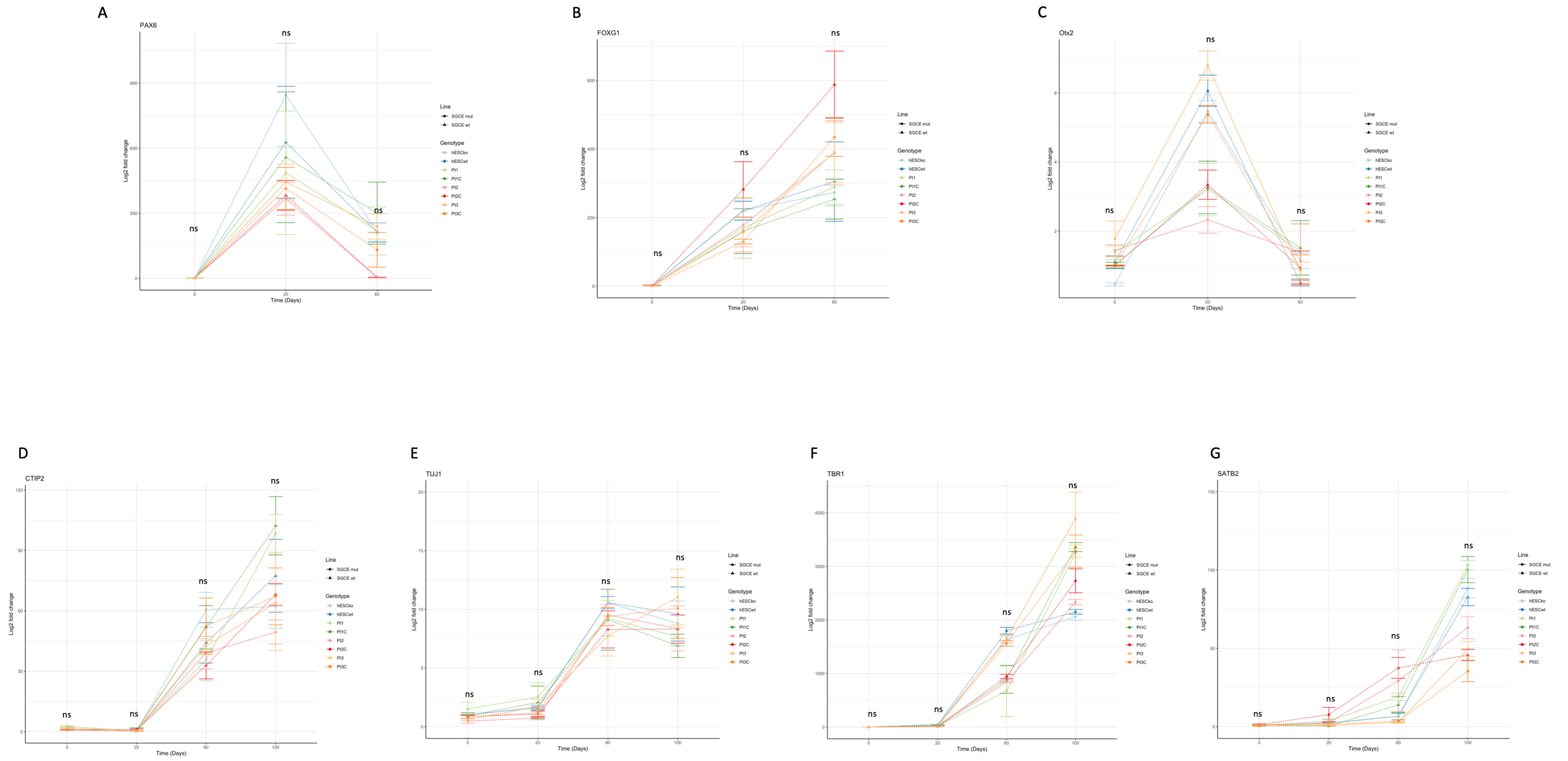

Figure S3: A-C: qRT-PCR expression at Days 0, 20 and 60 for multiple cortical markers (A) PAX6, (B) FOXG1, (C) Otx2. Each *SGCE* mutation carrying line is compared to their wild-type isogenic control. Data presented as mean±SEM from 3 independent experiments per line. Lines compared using two-way ANOVA analysis, with *p<0.05. **p<0.01, ***p<0.001, ns: not significant.

D-G: qRT-PCR expression at Days 0, 20, 60 and 100 for multiple cortical markers (D) CTIP2, (E) TUJ1, (F) TBR1, (G) SATB2. Each *SGCE* mutation carrying line is compared to their wild-type isogenic control. Data presented as mean±SEM from 3 independent experiments per line. Lines compared using two-way ANOVA analysis, with *p<0.05. **p<0.01, ***p<0.001, ns: not significant.

Supplementary Figure 4 (Figure S4)

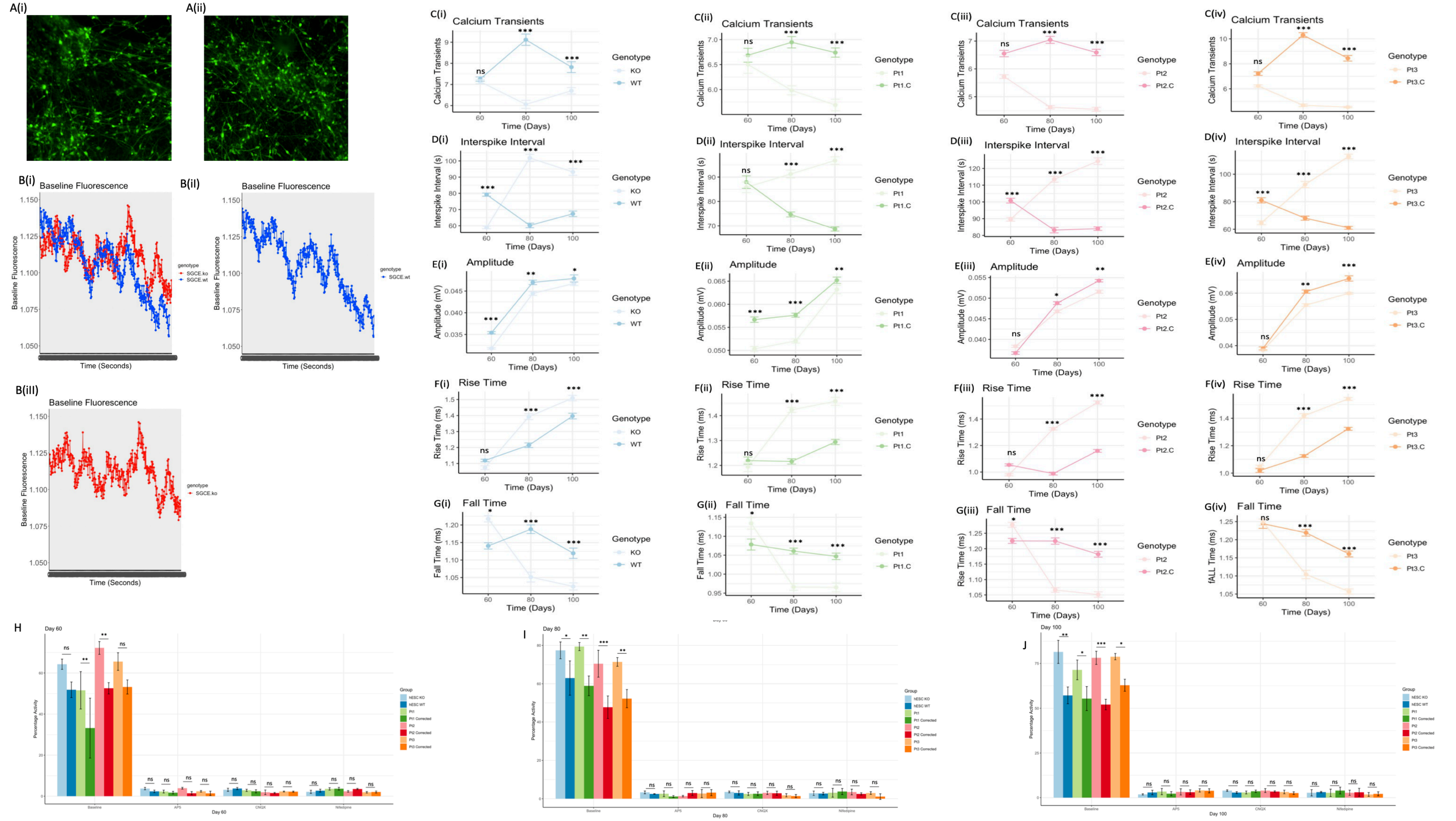

Figure S4: A: Representative image of Ca^{2+} recordings in D80 cells loaded with Flu0-4AM calcium indicator (wild-type(i) and *SGCE*-mutation positive(ii), B: Representative traces of spontaneous Ca2+ peaks, combined wild-type and *SGCE*-mutation positive (i), individual wild-type (ii) and *SGCE*-mutation positive (iii) **C-G:** Line plots depicting the change in the number of calcium transients (Ai-iv), Interspike Interval (Bi-iv), Amplitude (Ci-iv), Rise Time (Di-iv) and Fall Time (Ei-iv) over time. H-J: Calcium activity following application to the pharmacological inhibitors AP5, CNQX and nifedipine in comparison to baseline activity measurements at D60 (H), D80 (I) and D100 (J). Data presented as mean±SEM from 3 independent experiments per line. Statistical comparison using two-way ANOVA analysis. ns: not significant, *p<0.05, **p<0.01, ***p<0.001.

Supplementary Figure 5 (Figure S5)

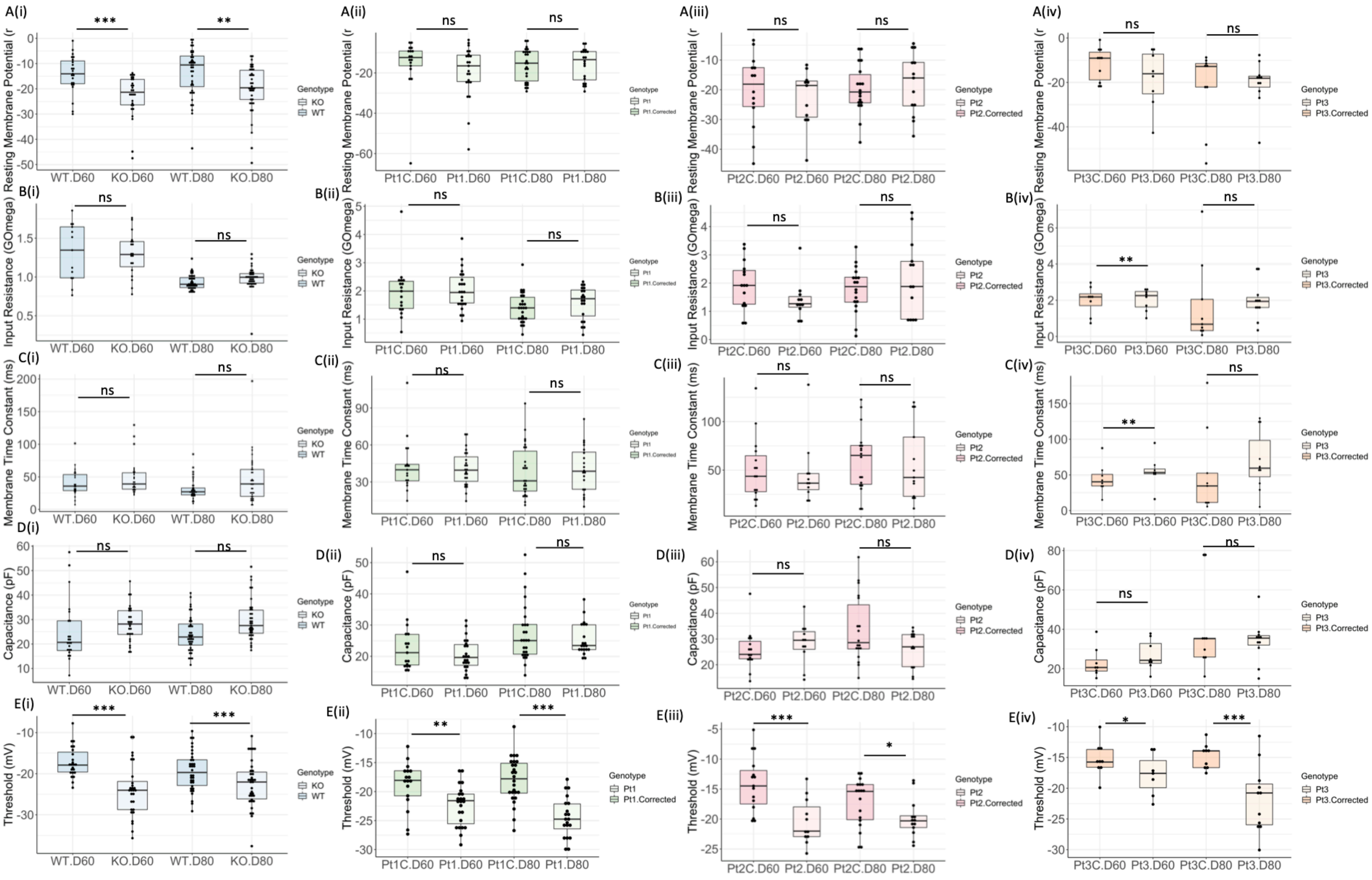

Figure S5: A-E: Box plots demonstrating baseline neuronal membrane properties across all paired cell lines at D60 and D80 timepoints. These include Resting Membrane Potential (Fi-iv), Input Resistance (Gi-iv), Membrane Time Constant (Hi-iv) and Capacitance (Ii-iv) and action potential threshold (Ji-iv).

Supplementary Figure 6 (Figure S6)

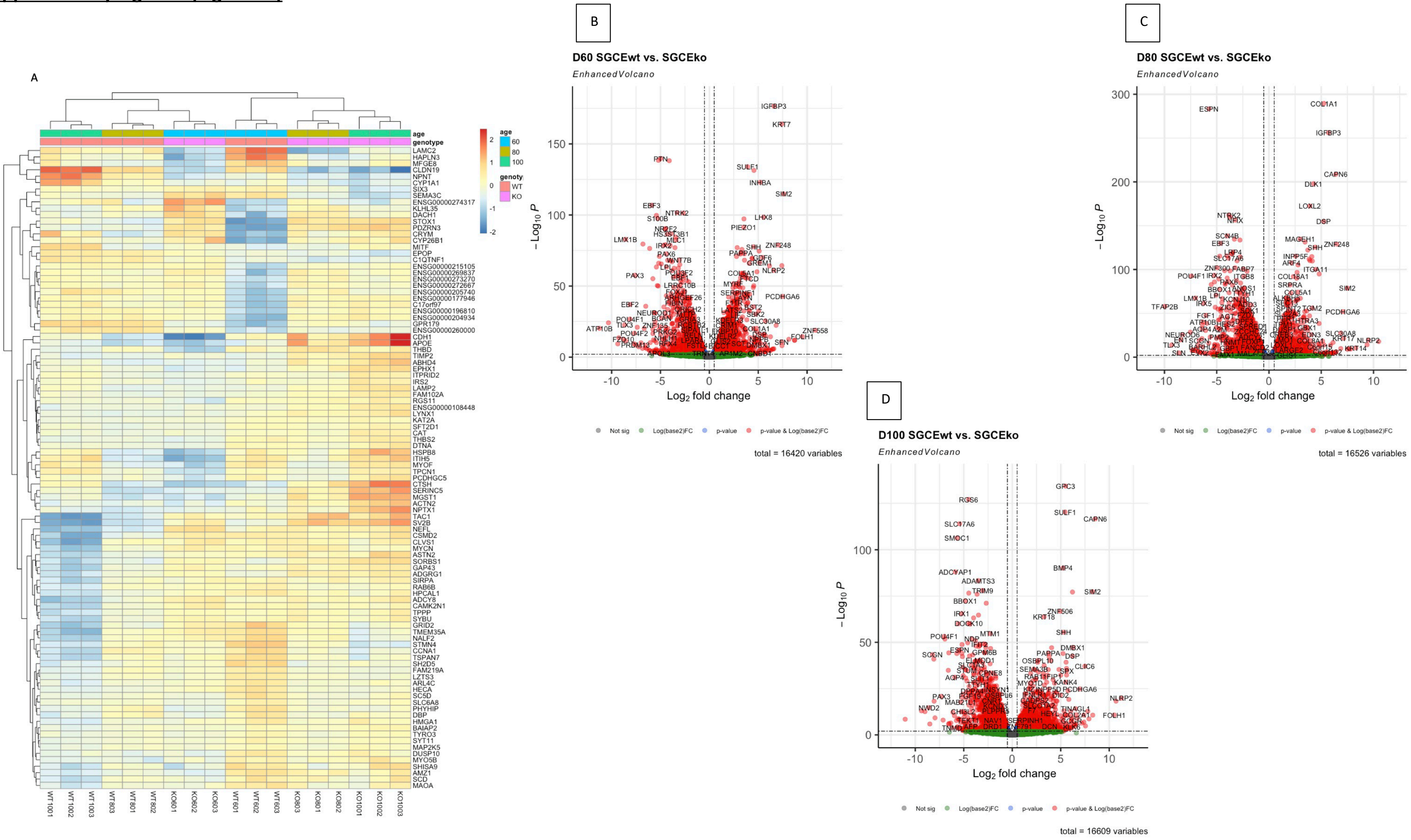

Figure S6: A: Heat map showing hierarchical clustering of protein-coding Differentially Expressed Genes (DEGs) in gene edited *SGCE*ko hESC compared to wild-type controls (*SGCE*wt) at time points; D60, D80, D100 (n=3 at each time point, for each line). **B-D:** Volcano plots demonstrating the number of significantly different and non-significantly differentially expressed genes at D60 (B), D80 (C) and D100 (D).

Supplementary Figure 7 (Figure S7)

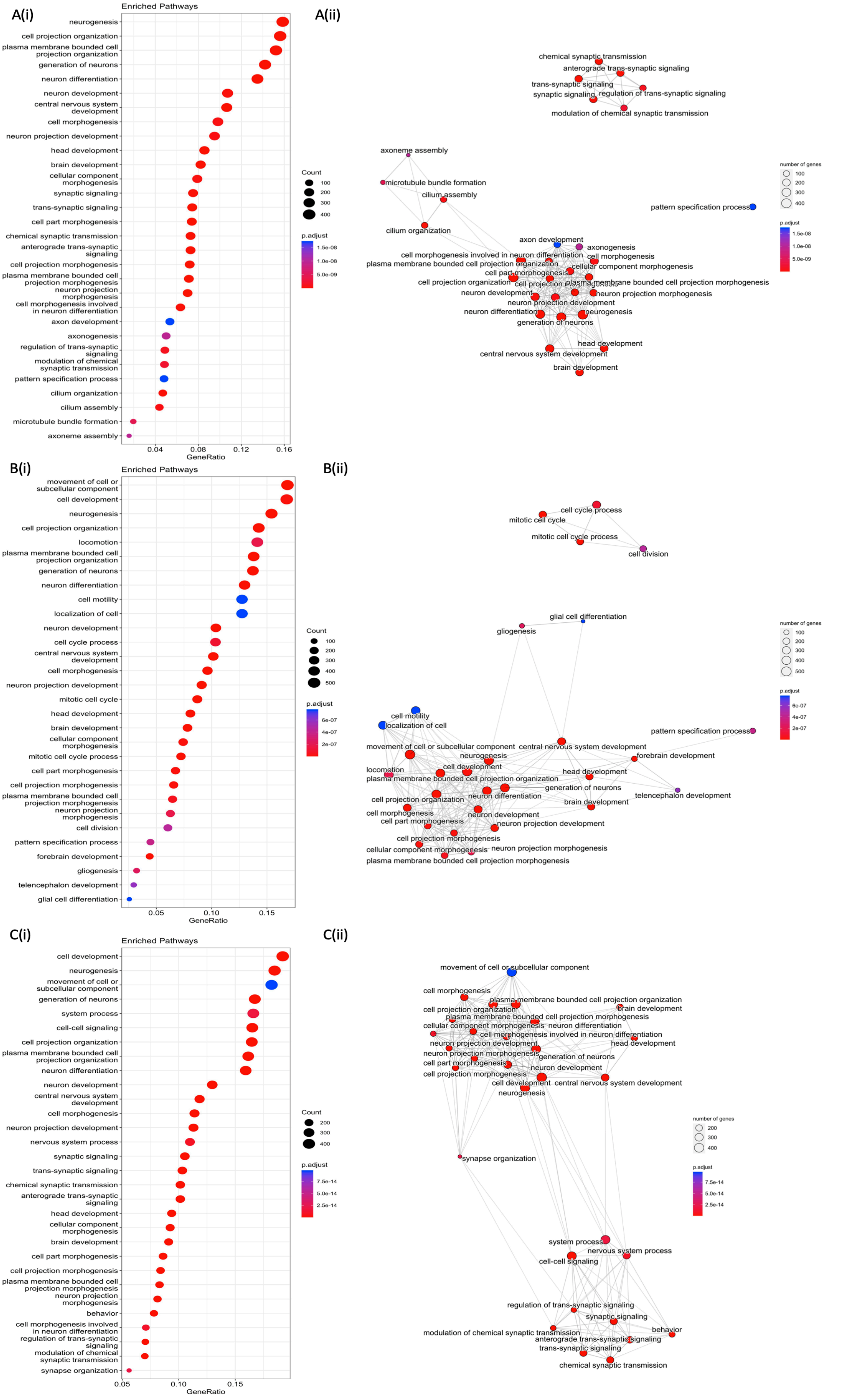

Figure S7: Bulk RNA-seq gene set enrichment analysis of upregulated genes in *SGCE*ko compared to *SGCE*wt lines at days 60, 80 and 100. Dot plot representation of the top overexpressed 30 gene ontology (GO) terms in *SGCE*ko line compared to *SGCE*wt at D60 **(Ai)**, D80 **(Bi)**, D100 **(Ci)**. GO terms are sorted by their adjusted p-value with Banjamini-Hochberg P-value correction of False Discovery Rate (FDR) <0.05. GO term enrichment analysis of the top 30 categories presented as enrichment maps for *SGCE*ko: *SGCE*wt comparison at D60 **(Aii)**, D80 **(Bii)**, D100 **(Cii)**. GO functional groups exhibiting higher statistically significant differences using Benjamini-Hochberg p-value correction (FDR<0.05) are shown. Network graph nodes represent GO terms (the most significant are named), and edges indicate shared genes between GO terms. Within this the nodes represent gene-sets, and the edges represent mutual overlap, such that highly redundant gene sets are grouped together as clusters, enabling visual interpretation of these relationships

Supplementary Figure 8 (Figure S8)

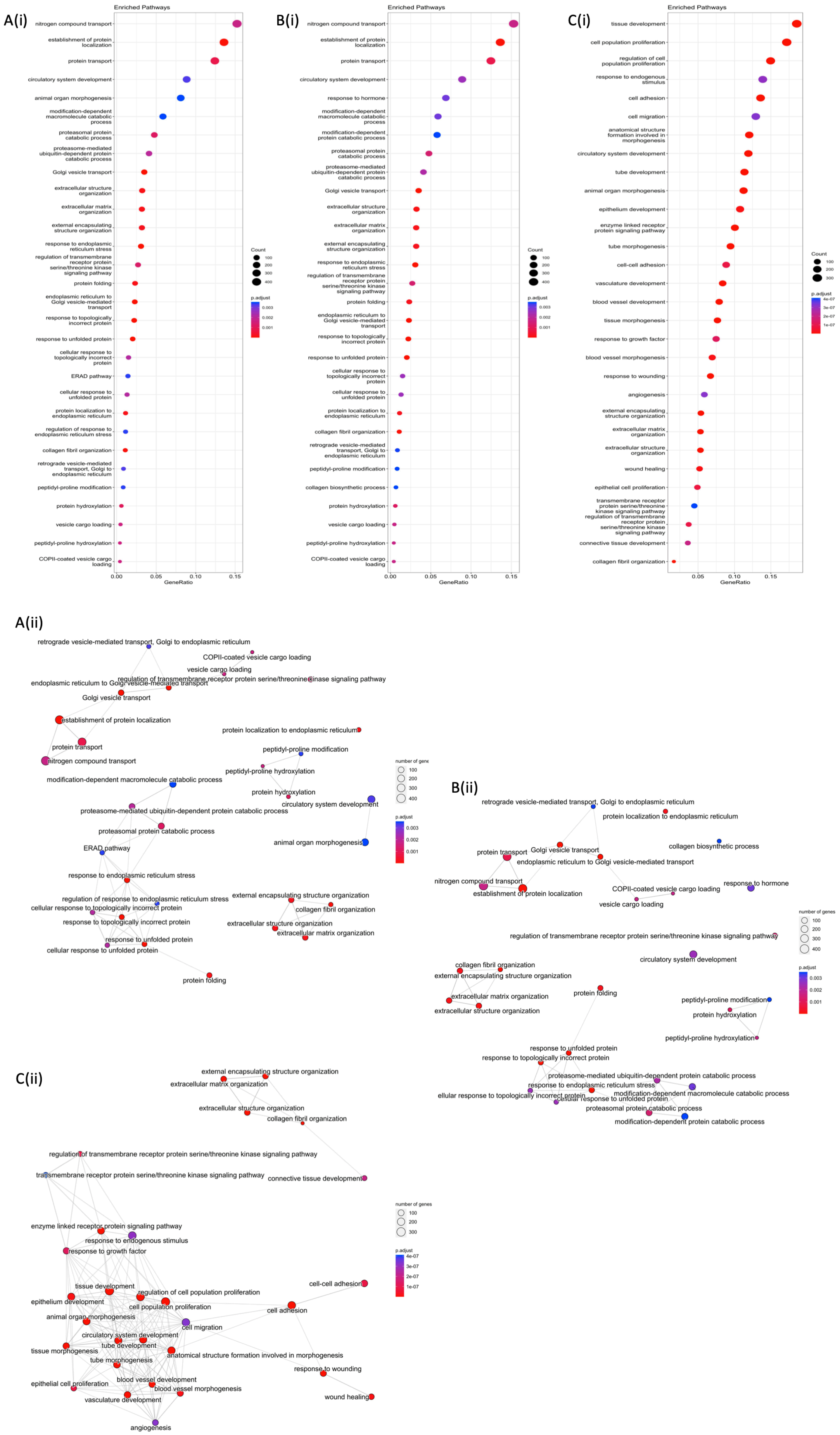

Figure S8: Bulk RNA-seq gene set enrichment analysis of downregulated genes in *SGCE*ko compared to *SGCE*wt lines at days 60, 80 and 100. Dot plot representation of the top overexpressed 30 gene ontology (GO) terms in *SGCE*ko line compared to *SGCE*wt at D60 **(Ai)**, D80 **(Bi)**, D100 **(Ci)**. GO terms are sorted by their adjusted p-value with Banjamini-Hochberg P-value correction of False Discovery Rate (FDR) <0.05. GO term enrichment analysis of the top 30 categories presented as enrichment maps for *SGCE*ko: *SGCE*wt comparison at D60 **(Aii)**, D80 **(Bii)**, D100 **(Cii)**. GO functional groups exhibiting higher statistically significant differences using Benjamini-Hochberg p-value correction (FDR<0.05) are shown. Network graph nodes represent GO terms (the most significant are named), and edges indicate shared genes between GO terms. Within this the nodes represent gene-sets, and the edges represent mutual overlap, such that highly redundant gene sets are grouped together as clusters, enabling visual interpretation of these relationships

Supplementary Figure 9 (Figure S9)

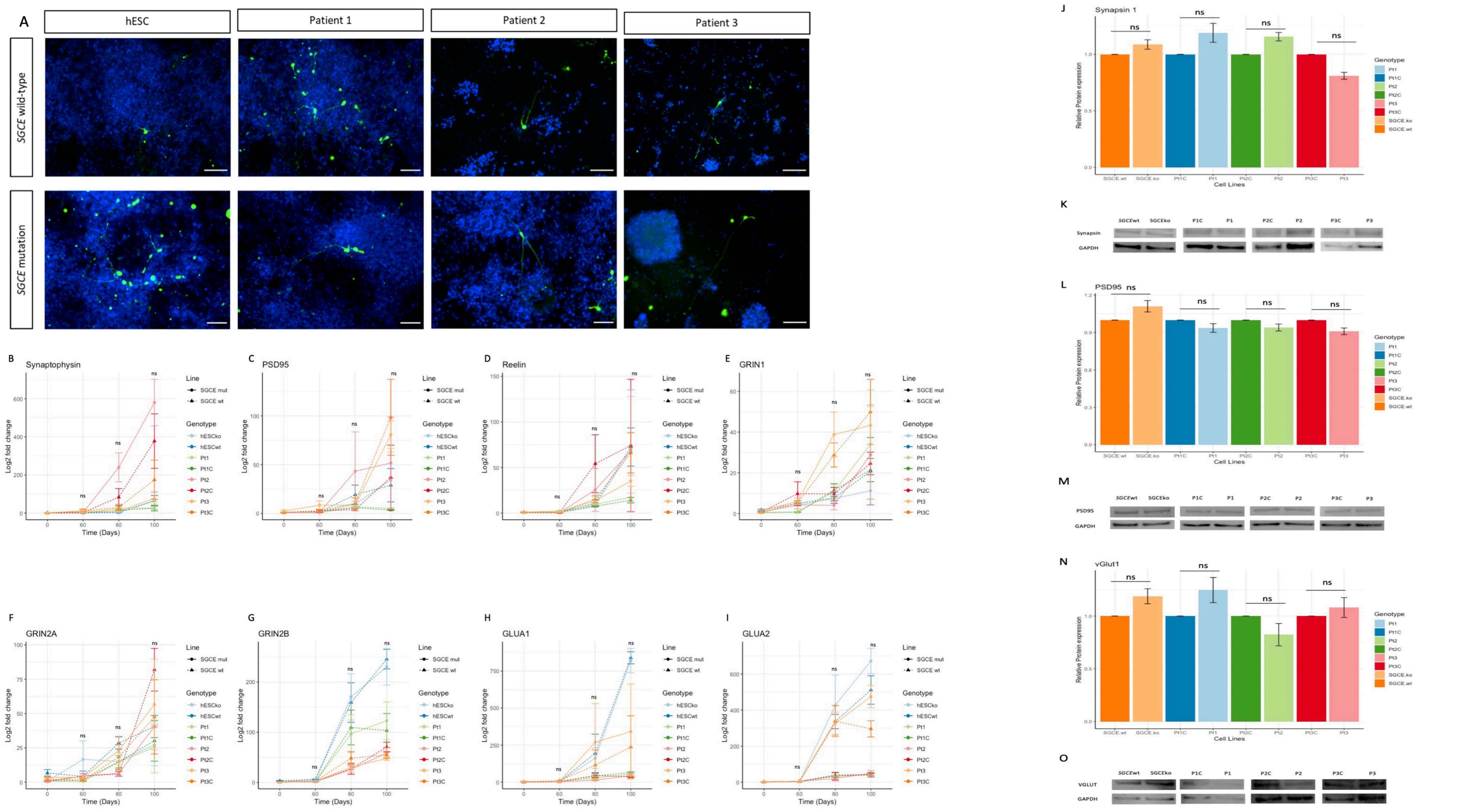

Figure S9: **A:** Representative images of GFP expressing cells counterstained with the nuclear marker DAPI, **B-I:** qRT-PCR expression at Days 0, 60, 80 and 100 for multiple synaptic markers. Each *SGCE* mutation carrying line is compared to their wild-type isogenic control. Data presented as mean±SEM from 3 independent experiments per line. Lines compared using two-way ANOVA analysis, with *p<0.05. **p<0.01, ***p<0.001, ns: not significant. Synaptic markers include: (A) Synaptophysin, (B) PSD95, (C) Reelin, (D) GRIN1, (E) GRIN2A, (F) GRIN2B, (G) GLUA1, (H) GLUA2. **J:** Quantification of relative Synapsin abundance in total neuronal cell lysates. (n=3 for all). Data presented as mean±SEM. Statistical comparison with Student's T-test. *p<0.05. **p<0.01, ***p<0.001, ns: not significant, **K:** Representative immunoblot for Synapsin and loading control (GAPDH), **L:** Quantification of relative PSD95 abundance in total neuronal cell lysates. (n=3 for all). Data presented as mean±SEM. Statistical comparison with Student's T-test. *p<0.05. **p<0.01, ***p<0.001, ns: not significant, **M:** Representative immunoblot for PSD95 and loading control (GAPDH), **N:** Quantification of relative VGLUT1 abundance in total neuronal cell lysates. (n=3 for all). Data presented as mean±SEM. Statistical comparison with Student's T-test. *p<0.05. **p<0.01, ***p<0.001, ns: not significant, **O:** Representative immunoblot for VGLUT1 and loading control (GAPDH)

Supplementary Methods Table 1: List of antibodies

Antibodies	Concentration	Source	Identifier
Mouse monoclonal anti-Ankyrin-G	1:500	UC Davis/NIH NeuroMab	Cat# N106/36; RRID: AB_2877524
Rabbit monoclonal anti-Brachyury	1:1000	Abcam	Cat# ab209665; RRID: EPR18113
Rat monoclonal anti-CTIP2	1:500	Abcam	Cat# ab18465; RRID: AB_2064130
Rabbit polyclonal anti-FOXG1,	1:250	Abcam	Cat# ab18259; RRID: AB_732415
Mouse monoclonal anti-KI67	1:1000	Leica Biosystems	Cat# ACK02
Goat polyclonal anti-GFP	1:500	R&D	Cat# AF4240; RRID: AB_884445
Mouse monoclonal anti-MAP2	1:500	Sigma-Aldrich	Cat# M1406; RRID: AB_477171
Rabbit polyclonal anti-MAP2	1:500	Millipore	Cat# AB5622; RRID: AB_91939
Mouse monoclonal anti-NESTIN	1:300	BD Pharmigen	Cat# 611659; RRID: 399177
Mouse monoclonal anti-NEUN	1:500	Millipore	Cat# MAB377; RRID: AB_2298772
Rabbit polyclonal anti-NEUN	1:250	Millipore	Cat# ABN78; RRID: AB_10807945
Rabbit polyclonal anti-pan-Neurexin-1	1:1000	Millipore	Cat# ABN161; RRID: AB_10917110
Rabbit polyclonal anti-Neuroligin-1	1:1000	Synaptic Systems	Cat# 129013; RRID: AB_2151646
Goat polyclonal anti-Neuroligin-4	1:1000	Synaptic Systems	Cat# EB11592; RRID: AB_2801590
Goat polyclonal anti-OCT3/4	1:500	Santa Cruz	Cat# SC8628; RRID: AB_653551
Rabbit anti-OTX2	1:300	Millipore	Cat# ab9566; RRID: AB_215186
Mouse monoclonal anti-PAX6	1:1000	DSHB	Cat# pax6; RRID: AB_528427
Mouse monoclonal anti-PSD95	1:1000	Thermo Fisher Scientific	Cat# MA1-046; RRID: AB_2092361
Rabbit monoclonal anti-Synapsin-1	1:1000	Cell Signalling Technology	Cat# 5297; RRID: AB_2616578
Rabbit monoclonal anti-TBR1	1:500	Abcam	Cat# ab31940; RRID: AB_2200219
Rabbit polyclonal anti-TBR2/Eomes	1:500	Abcam	Cat# ab23345; RRID: AB_778267
Mouse monoclonal anti-TRA-1-81	1:100	BD Pharmigen	Cat# 560072; RRID: AB_1645605
Mouse monoclonal anti-TUJ1	1:400	Sigma-Aldrich	Cat# 560072; RRID: AB_2315519
Rabbit polyclonal anti-VGLUT1	1:1000	Synaptic Systems	Cat# 135303; RRID: AB_887875

Supplementary Methods Table 2: Primer List

**gRNA sequence for iCas9 CRISPR editing**		
Exon 2: CRISPR site 1	TCTCCAAGGTACACTCCGAT	
Exon 2: CRISPR site 2	AGGTACACTCCGATCGGAAT	
Exon 2: CRISPR site 4	CGGAATGTATACCCATCAGC	
**Primer sequences for *SGCE* CRISPR site**		
	**Forward Sequence**	**Reverse Sequence**
SGCE CRISPR site	AGGGCGTATCTCATTATTTGTCT	TCCACCTTACCCAAAACCTG
**qPCR primer sequences**		
	**Forward Sequence**	**Reverse Sequence**
β-ACTIN	TCACCACCACGGCCGAGCG	TCTCCTTCTGCATCCTGTCG
SGCE	GGAGATTTTCGTTTGACAAC	CAACATGCATAACATATGCCAG
Cdc42	CAAGGACATTTGTTTGCCATT	TTTGGTGCATTTCAAAGGTG
CTIP2	CTCCGAGCTCAGGAAAGTGTC	TCATCTTTACCTGCAATGTTCTCC
EMX2	GCTTCTAAGGCTGGAACACG	CCAGCTTCTGCCTTTTGAAC
FOXG1	TGGCCCATGTCGCCCTTCCT	GCCGACGTGGTGCCGTTGTA
GAPDH	ATGACATCAAGAAGGTGGTG	CATACCAGGAAATGAGCTTG
Nestin	AGCAGGAGAAACAGGGCCTAC	CTCTGGGGTCCTAGGGAATTG
OTX2	TGCCAAAAAGAAGACATCTCCA	AAGCTGGGCTCCAGATAGACAC
PAX6	AACAGACACAGCCCTCACAAACA	CGGGAACTTGAACTGGAACTGAC
PSD95	TCCACTCTGACAGTGAGACCGA	CGTCACTGTCTCGTAGCTCAGA
Reelin	ATGTGGAGGTCGTCCTAGTAAGC	GGAAAGTGGTGTACACTCGG
SATB2	CAACGCAACTAATAATCATCTCCC	GAGAAAGGGCTGAGAACCCG
Synaptophysin	TCGGCTTTGTGAAGGTGCTGCA	TCACTCTCGGTCTTGTTGGCAC
TUJ1	CATGGACAGTGTCCGCTCAG	CAGGCAGTCGCAGTTTTCAC
vGlut1	GAAACTCATGAACCCCCTCA	GGGAGATGAGCAGCAGGTAG
vGlut2	ATTCCATCAGCAGCCAGAGT	TTGCTCCATATCCCATGACA
