# Supplementary material for: Cortical neuronal hyperexcitability and synaptic changes in SGCE mutation-positive myoclonus dystonia
Source: Brain. 2022 Oct 7;146(4):1523–41. doi: 10.1093/brain/awac365 (PMC10115238; doi:10.1093/brain/awac365)

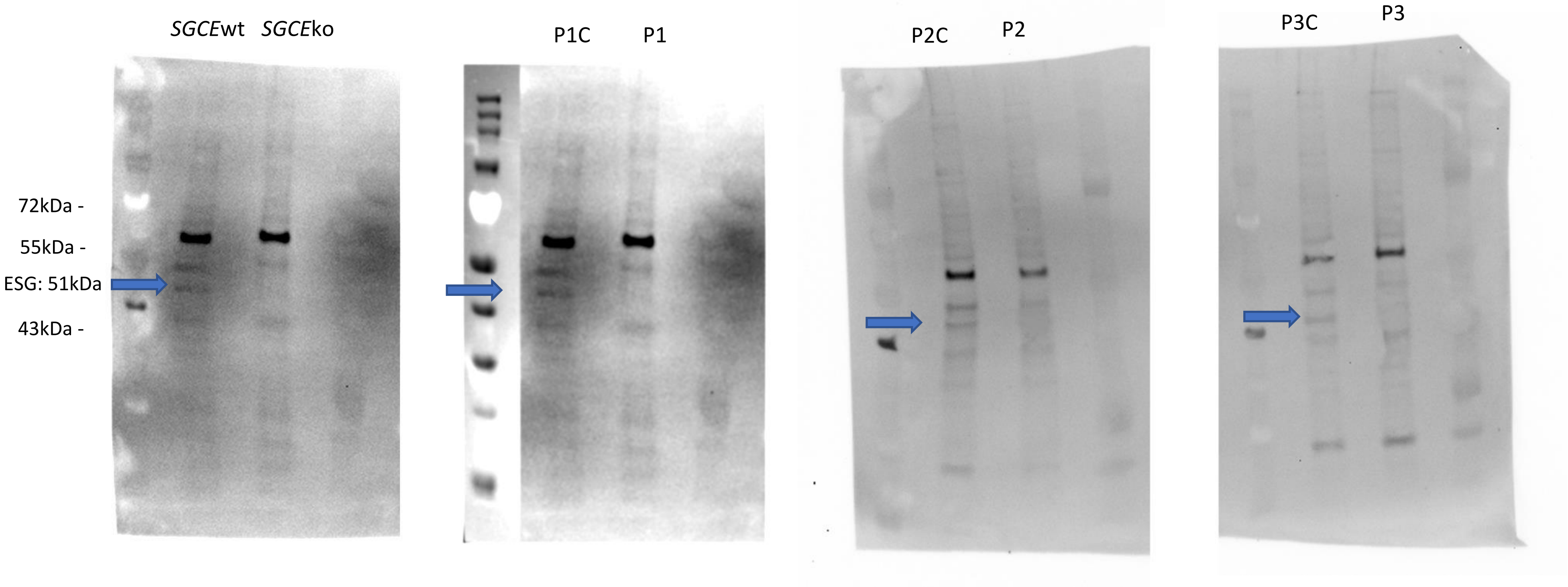
SGCEwt
SGCEko
P1C
P1
P2C
P2
P3C
P3
72kDa -
55kDa -
ESG: 51kDa
43kDa -

Figure S9: K (Synapsin)

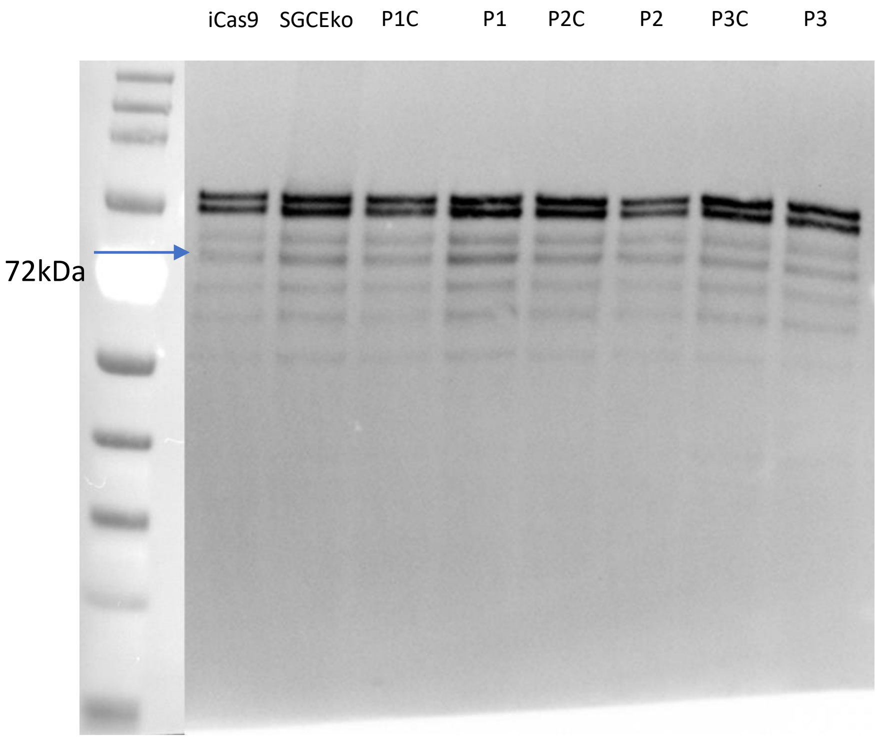

Figure S9: M (PSD95)

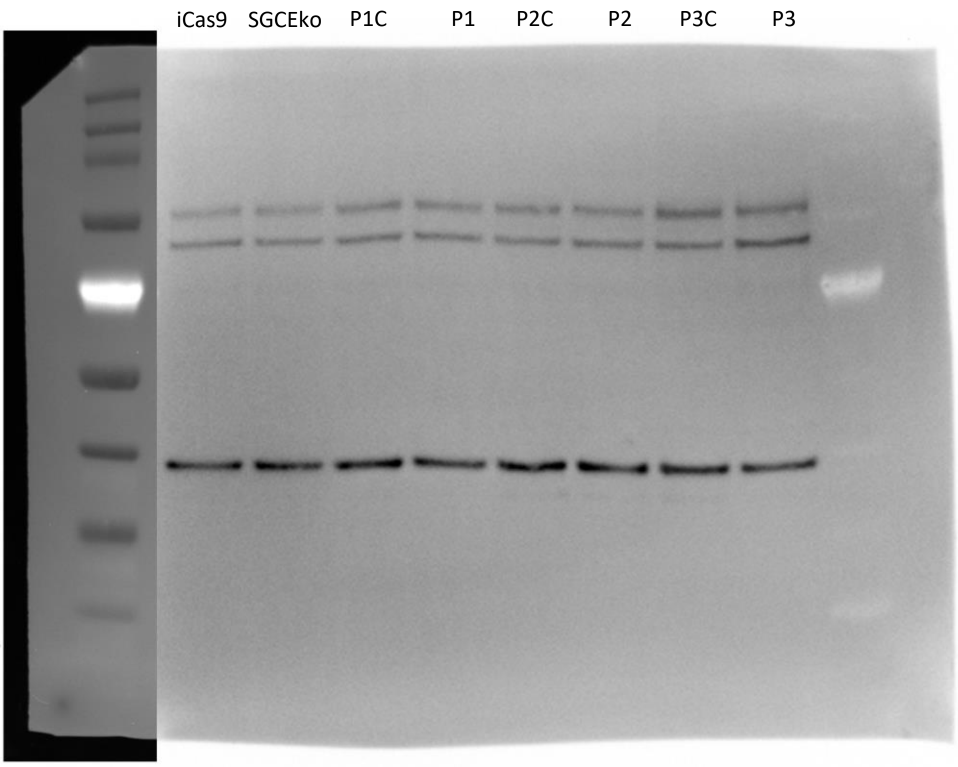

Figure S9: O (VGLUT1)

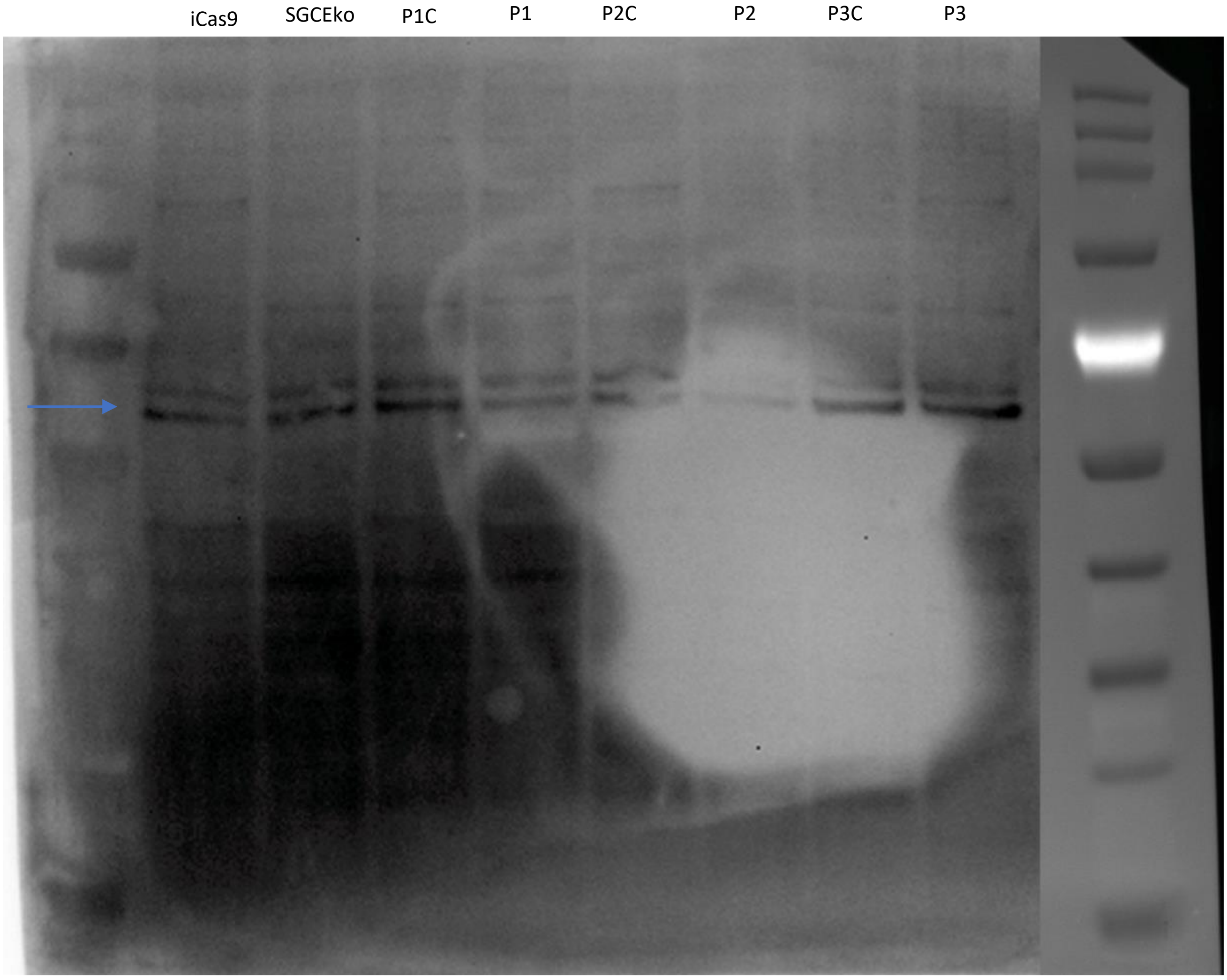

Figure 5: E (Neuroligin-4)

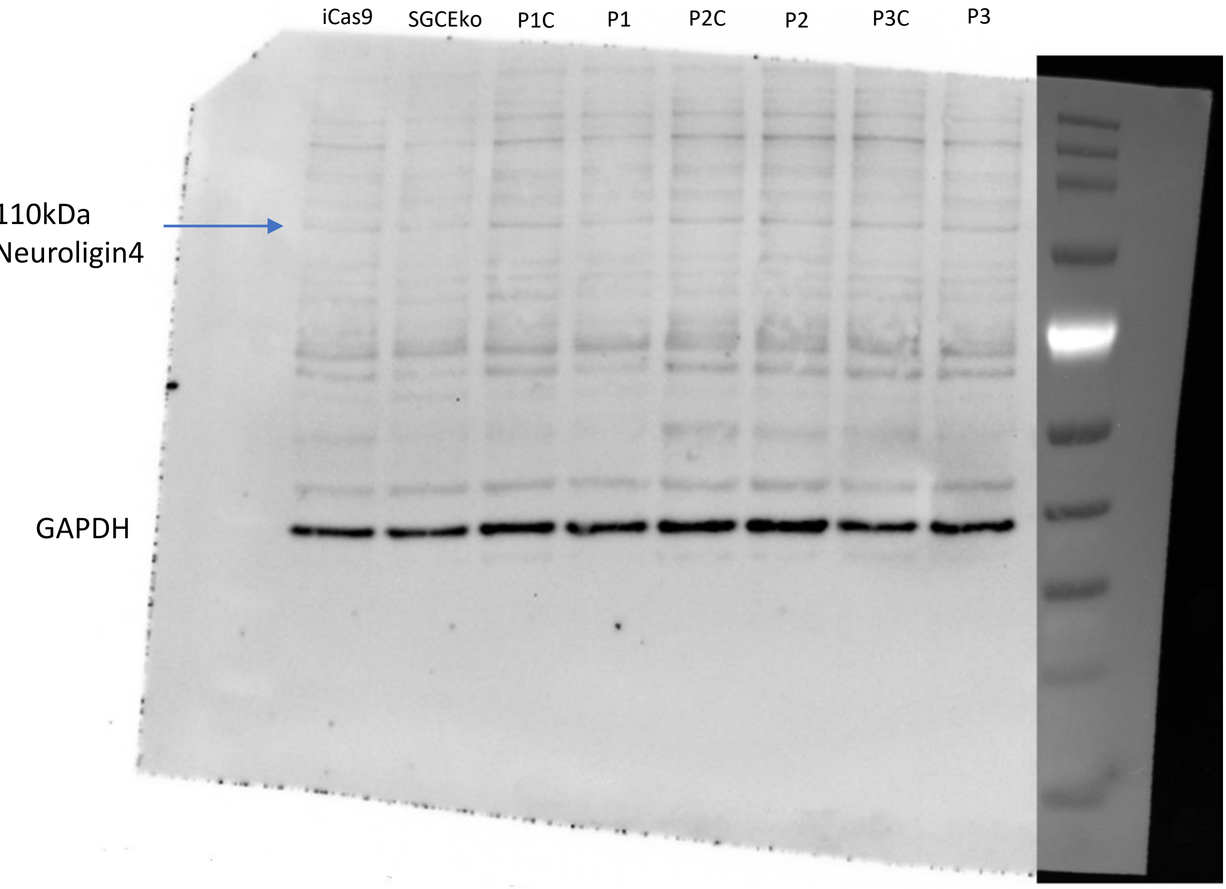

Figure 5: H (Neurexin-1)

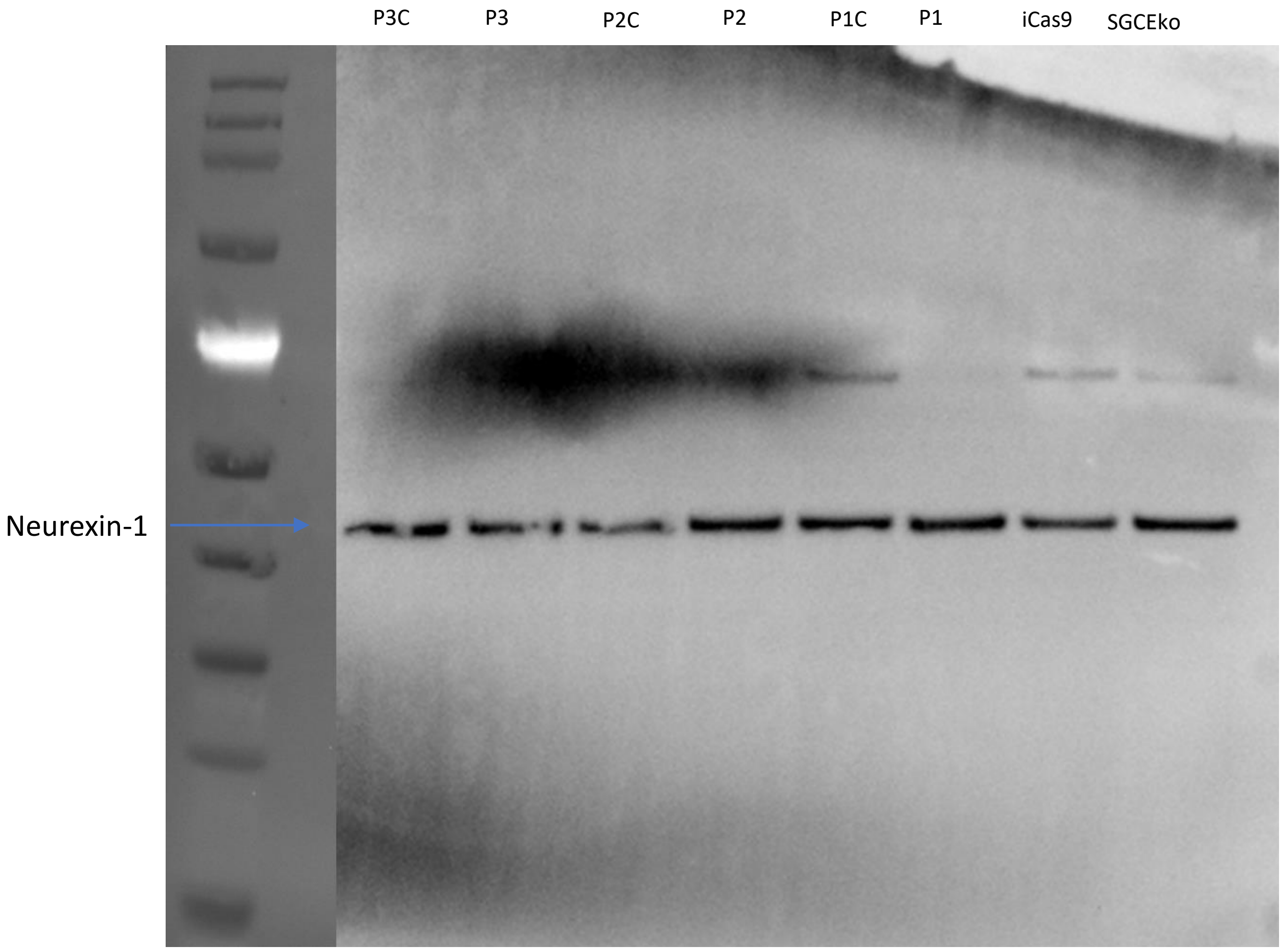

Supplement: awac365_Supplementary_Data [file awac365_supplementary_data.zip › brain-2022-00185-File018.pdf]
